# European Forest Cover During the Past 12,000 Years: A Palynological Reconstruction Based on Modern Analogs and Remote Sensing

**DOI:** 10.3389/fpls.2018.00253

**Published:** 2018-03-08

**Authors:** Marco Zanon, Basil A. S. Davis, Laurent Marquer, Simon Brewer, Jed O. Kaplan

**Affiliations:** ^1^Institute of Pre- and Protohistoric Archaeology, Christian-Albrechts-Universität zu Kiel, Kiel, Germany; ^2^Graduate School “Human Development in Landscapes”, Christian-Albrechts-Universität zu Kiel, Kiel, Germany; ^3^Institute of Earth Surface Dynamics, University of Lausanne, Lausanne, Switzerland; ^4^Department of Physical Geography and Ecosystem Science, Lund University, Lund, Sweden; ^5^GEODE, UMR-CNRS 5602, Université de Toulouse-Jean Jaurès, Toulouse, France; ^6^Research Group for Terrestrial Palaeoclimates, Max Planck Institute for Chemistry, Mainz, Germany; ^7^Department of Geography, University of Utah, Salt Lake City, UT, United States; ^8^ARVE Research SARL, Pully, Switzerland; ^9^Department of Archaeology, Max Planck Institute for the Science of Human History, Jena, Germany

**Keywords:** Modern Analog Technique, forest cover, pollen data, remote sensing, Europe, Younger Dryas, Holocene

## Abstract

Characterization of land cover change in the past is fundamental to understand the evolution and present state of the Earth system, the amount of carbon and nutrient stocks in terrestrial ecosystems, and the role played by land-atmosphere interactions in influencing climate. The estimation of land cover changes using palynology is a mature field, as thousands of sites in Europe have been investigated over the last century. Nonetheless, a quantitative land cover reconstruction at a continental scale has been largely missing. Here, we present a series of maps detailing the evolution of European forest cover during last 12,000 years. Our reconstructions are based on the Modern Analog Technique (MAT): a calibration dataset is built by coupling modern pollen samples with the corresponding satellite-based forest-cover data. Fossil reconstructions are then performed by assigning to every fossil sample the average forest cover of its closest modern analogs. The occurrence of fossil pollen assemblages with no counterparts in modern vegetation represents a known limit of analog-based methods. To lessen the influence of no-analog situations, pollen taxa were converted into plant functional types prior to running the MAT algorithm. We then interpolate site-specific reconstructions for each timeslice using a four-dimensional gridding procedure to create continuous gridded maps at a continental scale. The performance of the MAT is compared against methodologically independent forest-cover reconstructions produced using the REVEALS method. MAT and REVEALS estimates are most of the time in good agreement at a trend level, yet MAT regularly underestimates the occurrence of densely forested situations, requiring the application of a bias correction procedure. The calibrated MAT-based maps draw a coherent picture of the establishment of forests in Europe in the Early Holocene with the greatest forest-cover fractions reconstructed between ∼8,500 and 6,000 calibrated years BP. This forest maximum is followed by a general decline in all parts of the continent, likely as a result of anthropogenic deforestation. The continuous spatial and temporal nature of our reconstruction, its continental coverage, and gridded format make it suitable for climate, hydrological, and biogeochemical modeling, among other uses.

## Introduction

Determining the spatial structure of land cover and its variation through time is essential in order to understand the interplay between biosphere, atmosphere, and human societies. Knowledge of past vegetation dynamics is of great interest to a range of disciplines dealing with landscape, climate, human development, and their reciprocal interactions. For example, information concerning past forest cover influences the archeological narrative (e.g., Kreuz, 2007) and plays a tangible role in defining modern management strategies (Vera, 2000; Mitchell, 2005; Bradshaw et al., 2015); vegetation cover data are a central component of Earth system models dealing with carbon storage and release (e.g., Kaplan et al., 2002) and for investigating the feedbacks between land cover and climate (Gaillard et al., 2010); similarly, the simulation of past human–plants interactions is needed for the understanding of human imprint on ecosystems from early history up to the present day (Pongratz et al., 2008; Kaplan et al., 2009).

Recent landscape history makes use of different mapping technologies, ranging from historical and cartographic sources (e.g., Hohensinner et al., 2013) to satellite imagery (Bossard et al., 2000; DeFries et al., 2000; Hansen et al., 2013). Beyond the reach of these mapping means, our ability to infer past land cover depends on the interpretation of paleoenvironmental proxies. Past landscape information has been extracted from different archives, such as fossil pollen (e.g., Edwards et al., 2017), plant macrofossils (e.g., tree line studies; Nicolussi et al., 2005), mollusks (e.g., Preece et al., 1986), beetles (e.g., Whitehouse and Smith, 2004), biochemical tracers (e.g., McDuffee et al., 2004) and ancient DNA (Birks and Birks, 2016). Among the diverse sources available, each offering a different point of view on similar questions, the analysis of pollen grains remains unsurpassed in terms of spatial and temporal coverage. The simplest approach in palynology consists of using arboreal versus non-arboreal pollen percentages to estimate forest cover, and in using indicator species (e.g., Behre, 1981) to infer changes in land use. The growing availability of pollen archives at a continental scale has then made it possible to track species expansion/extinction based on isolines and threshold values (e.g., Huntley and Birks, 1983; Brewer et al., 2002, 2016; Ravazzi, 2002; Finsinger et al., 2006). These approaches can be qualified as purely qualitative, as the non-linear relationship between plant abundances and pollen percentages is acknowledged but not corrected for (Gaillard et al., 2008).

Semi-quantitative models have been developed by grouping individual taxa into plant functional types (PFTs) and biomes (Prentice et al., 1996; Peyron et al., 1998), thus providing a consistent methodology to distinguish major vegetation types. The biomisation approach is able to recognize boundaries between plant communities (Williams et al., 2000), although difficulties have emerged at ecotones such as the forest-steppe boundary due to a bias in the method toward arboreal taxa. This bias arose from the original objective of the method to replicate potential natural vegetation (Peyron et al., 1998; Tarasov et al., 1998), and therefore to minimize the role of non-arboreal taxa symptomatic of anthropogenically deforested landscapes. A further problem has been the categorical nature of the biome assignment, leading to difficulties in recognizing gradual ecotonal transitions and producing spatially continuous reconstructions. Often the results have been mapped as point estimates (e.g., Prentice et al., 2000). Continuous spatial fields have been attempted using different techniques, such as in Peng et al. (1995), Collins et al. (2012), and Fyfe et al. (2015). While biomes are distinguished into forest and non-forest types, Collins et al. (2012) also used forest and non-forest PFTs derived from the same biomisation formulae as a more continuous measure of forest cover.

A first attempt to achieve a real quantification was pioneered by Prentice and Parsons (1983), Prentice (1985), and Sugita (1993, 1994), gradually evolving into the Landscape Reconstruction Algorithm (LRA). The LRA models – these are REVEALS for regional plant abundance and LOVE for local plant abundance (Sugita, 2007a,b) – make use of a comprehensive set of parameters (region-specific pollen productivity estimates, fall speed of pollen, basin size) and assumptions (e.g., wind speed and direction, atmospheric conditions) to simulate pollen dispersal/deposition mechanisms and decrease biases deriving from the non-linear relationships between plant abundances and pollen data.

Pollen-based vegetation reconstructions produced with the LRA are currently available for most of Europe outside of the Mediterranean (e.g., Nielsen and Odgaard, 2010; Sugita et al., 2010; Nielsen et al., 2012; Cui et al., 2013, 2014; Fyfe et al., 2013; Overballe-Petersen et al., 2013; Marquer et al., 2014, 2017; Mazier et al., 2015; Trondman et al., 2015). A wider application of the method to the Mediterranean depends on the collection of reliable pollen productivity estimates for the region, which are not currently available. A different quantitative approach, not requiring pollen productivity estimates demanded by the LRA method, was developed and applied in North America by Williams (2002) and Williams and Jackson (2003). This approach uses instead the Modern Analog Technique (MAT), based on the basic assumption that pollen samples sharing a similar composition are the by-product of comparable vegetation assemblages. Therefore, given two pollen samples – a modern and a fossil one – composed by a similar mixture of taxa, the environmental parameters of the first (e.g., forest cover, climate variables) can be directly transferred to the latter. The relationship between pollen assemblages and forest cover in the Williams method is established using a calibration dataset of modern pollen samples where the forest cover around the pollen site is estimated using satellite remote sensing from the Advanced Very High-Resolution Radiometer (AVHRR). Past forest cover is then reconstructed by assigning to each fossil sample the average forest cover of its closest modern analogs from amongst the modern samples in the calibration dataset. This approach was applied to Northern Eurasia (Tarasov et al., 2007; Kleinen et al., 2011) and to the whole forest-tundra ecotone of the Northern Hemisphere (Williams et al., 2011). Its application in Europe has been so far limited to point reconstructions for a few selected time windows (Williams et al., 2011).

In the present study, we test the capabilities of the MAT for continuous forest-cover reconstructions at a continental scale and from the Pleistocene/Holocene transition to the present day. We follow the methods used by Tarasov et al. (2007) and Williams et al. (2011) but with improved modern calibration and fossil datasets and spatially continuous mapping. The size of modern and fossil pollen data sets have been increased by 80 and 50%, respectively, compared to the European data used by Williams et al. (2011), while the quality of the metadata and chronological controls for the fossil data have also been greatly improved (Fyfe et al., 2009; Davis et al., 2013; Giesecke et al., 2013). The AVHRR-based forest-cover data (1 km resolution) used in all previous MAT applications has been upgraded with a 30-m resolution dataset (Hansen et al., 2013) based on LANDSAT.

Our results are presented here as both maps for the whole of Europe at 1,000-year intervals, and as regional area-average time-series. The full set of 49 maps, each one covering an ∼250-year interval, is available as Supplementary Material. Each map displays interpolated forest-cover data with continental coverage at a resolution of five arc-minutes. The predictive ability of our model was assessed though standard statistical indicators based on analysis of the modern training set [*r*^2^ and Root Mean Square Error of Prediction (RMSEP) from cross-validation exercises]. In addition, down core evaluation was also undertaken through a comparison between the MAT and the REVEALS-based forest-cover reconstructions for specific sites, thus testing the performance of our method against methodologically independent forest-cover reconstructions.

## Materials and Methods

### Fossil and Modern Pollen Data

The fossil pollen dataset used in our analysis is the same as presented in Mauri et al. (2015) and based largely on the European Pollen Database (EPD) with some additional data coming from the PANGAEA data archive^1^ and Collins et al. (2012) (Supplementary Figure S1). All age-depth models for all sites used calibrated ^14^C chronologies, with those for EPD sites based on the latest available chronologies from Giesecke et al. (2013).

The European Modern Pollen Database (EMPD; Davis et al., 2013) was selected as the source for modern palynological data (Supplementary Figure S2). The EMPD includes nearly 5,000 samples covering Eurasia and the circum-Mediterranean, which were filtered based on quality-control criteria (see section “Quality Filtering”).

Pollen spectra in both the modern and fossil datasets were converted into PFTs following the approach developed by Prentice et al. (1996) and refined by Peyron et al. (1998). Pollen taxa are grouped into PFTs following combinations of plant habit (e.g., woody, herbaceous), phenology (e.g., evergreen, deciduous), leaf form (e.g., broad-leaved, needle-leaved), and climatic range (Prentice, 1985). Differential pollen productivity and dispersal are not accounted for during PFT assignment. The main steps within the taxa-to-PFTs algorithm are presented in Supplementary Tables S1–S3. The full procedure is described in Peyron et al. (1998). The capabilities of PFTs in pollen-based climatic and ecological reconstructions have been tested in European contexts through the Holocene (Collins et al., 2012; Davis et al., 2015; Mauri et al., 2015), and were found preferable to taxa approaches in MAT-based climate reconstructions (Davis et al., 2003).

### Remote Sensing Data

Modern forest-cover values were extracted from the Global Forest Change dataset (Hansen et al., 2013), which includes estimates of forest-cover fraction for the year 2000. The forest-cover dataset produced by Hansen et al. (2013) was preferred over the AVHRR-based maps from DeFries et al. (2000) used in previous studies (Tarasov et al., 2007; Williams et al., 2011). While DeFries et al. (2000) offer a wider range of maps covering different vegetation categories, the dataset from Hansen et al. (2013) offers a much higher resolution and a wider range of values. DeFries et al. (2000) provide separate quantitative data on broadleaf, deciduous, evergreen, needle leaf, and total forest cover at a resolution of 1-km. Pixel values are continuous in the range 10–80% of forest cover (woody fraction), with specific codes to mark non-vegetated areas and areas with forest cover lower than 10%. In contrast, Hansen et al. (2013) offer a resolution of 1 arcsecond, equivalent to around 30-m at the equator, and a full range of pixel values from 0 to 100% canopy closure.

The average forest-cover values for the modern pollen data set were extracted by placing a search window around the location of each sample. We applied a general search radius of 50 km to approach a more consistent comparison between MAT and REVEALS estimates, because the REVEALS method suggests that its reconstructions are representative of a 50-km radius around the sites in question (see section “Comparison between MAT and REVEALS Estimates of Past forest cover”). A circular search window was preferred over a square one (used, e.g., in Williams et al., 2011), thus assuming a pollen source area with equal contributions from vegetation from all directions. The forest-cover value of every grid cell falling within the search radius was then distance-weighted using a two-dimensional Gaussian function centered on the pollen sample and subsequently averaged. A Gaussian curve was preferred primarily due to ease of implementation. Future model versions might include more “fat-tailed” distributions (e.g., Dawson et al., 2016). Given the predominance of moss polsters and soil samples in the EMPD, a value of σ = 500 m was used in order to assign a prevalent weight to the circular region immediately closer to the sample site (Supplementary Figure S3). A smaller σ value (100 m) was applied only to samples collected in densely forested areas (forest cover > 40% and EMPD sampling context described as “dense forest” or “forest undefined”). In these cases, the 100 m value was preferred over the 500 m one only when it yielded higher forest-cover values, suggesting the presence of denser forests right at the sampling location. A higher distance–weight parameter (σ = 10 km) was applied to samples collected from lakes in order to simulate a wider pollen source area. This σ-value is drawn from the optimal search window half-widths tested in previous studies (Williams and Jackson, 2003; Tarasov et al., 2007; Williams et al., 2011). Similarly to the procedure applied to moss and soil samples, a smaller σ-value (500 m) was applied to small water bodies (surface < 20 ha) surrounded by forested landscapes in order to emphasize the contribution of perilacustrine arboreal vegetation (Matthias and Giesecke, 2014). Since pixels occupied by water bodies were not included in the averaging procedure, weight is automatically added to the outer regions of the search window as both lake size and the number of pixels containing water around the core site increase. Bog sites therefore have the maximum weighting close to the core site. With lakes, the weight given to more distant pixels naturally increases as lake size increases and more pixels containing just water are excluded.

It should be stressed that pollen dispersal mechanisms vary largely from site to site depending on multiple factors (e.g., vegetation density, wind speed, pollen grains size and density, topography). Computational constraints, the wide diversity of the EMPD samples and, above all, the current availability of metadata prevent the calculation of sample-specific search windows and weight factors within this study. The application of narrow distance-weighting parameters (100 m and 500 m) was prompted by empirical and simulation studies on pollen deposition dynamics (e.g., Wright, 1952; Tinsley and Smith, 1974; Lavigne et al., 1996). Local foliage – as an example – might disrupt pollen dispersal, either by acting as a simple physical obstacle (Prentice, 1985) or via electrostatic capture (Bowker and Crenshaw, 2007), causing large amounts of pollen to reach the ground within few meters of the source. Sugita (1994) and Calcote (1995) estimate that between 20 and 60% of the pollen load in forest hollows comes from within a radius of < 100 m. Genetic analysis on plant communities appear to reach similar conclusions despite radically different methodological approaches. DNA paternity tests on *Pinus sylvestris* populations show that as much as ∼50% of the pollen reaching female cones may come from individuals located within a 10 m radius, and that more than 90% of the pollen-carried genetic material may come from within a radius of ∼200 m (Robledo-Arnuncio and Gil, 2005). When small *Quercus* sp. stands are considered (ca. ≤6 ha, equivalent – for comparative purposes – to a circular region with *r* = ∼140 m), between ∼30 and >80% of successful pollinations were ascribed to pollen coming from within the stand (Streiff et al., 1999; Gerber et al., 2014; Moracho et al., 2016). Surface pollen samples might therefore primarily record a signal from local/extra-local pollen sources (e.g., few dozens to several hundred meters, *sensu* Jacobson and Bradshaw, 1981). In the context of our study, a σ-value = 500 m is meant to stress the importance of nearby pollen sources, while a 100-m value points to a dominant contribution from local dense arboreal cover. Arguably, the LRA could be used to estimate the pollen contribution from vegetation at any given distance from a sample. This effort would at the very least require pollen productivity estimates for all taxa and regions involved in the present study, which are currently not available. As a consequence, the use of only three σ values (100 m, 500 m, and 10 km) represents a necessary simplification.

### Definition of Forest Cover

Study-specific definitions of forest cover should be kept in mind when interpreting and comparing the results from different quantitative approaches. REVEALS-based estimates of forest cover correspond to the overall regional tree abundance expressed in relative percentage cover. Age and size of trees are not assumed to be critical factors influencing the results, although they can have an influence on pollen productivity estimates and pollen dispersal and deposition (Jackson, 1994; Matthias et al., 2012; Baker et al., 2016). Analog-based approaches draw their definition of forest from their respective land cover data sets. Hansen et al. (2013) use a definition comparable to DeFries et al. (2000), describing forest-cover values per pixel as canopy closure percentages for all vegetation taller than 5 m. Significantly, DeFries et al. (2000) point out that a satellite-based mapping approach might identify shrubs as herbaceous vegetation if they are low to the ground, and as woody vegetation if they are taller. In an attempt to address this potential issue, we combined global vegetation height data (Simard et al., 2011) with the CORINE data set (Büttner et al., 2014) in order to detect areas occupied by forests but with a dominant tree height lower than 5 m. Modern samples with a median tree height = 0 m within the area covered by 1σ of the distance–weight function (i.e., the area surrounding the samples that contributes the most to tree cover quantification) were excluded from the analog pool. This filter was applied only to areas covered by the CORINE data set and only to lacustrine samples. The rationale behind the latter discrimination lies in our decision to prioritize the exclusion of easily discernible low-quality samples while at the same time limiting the occurrence of false positives. In this specific case, the relatively coarse resolution of the tree-height data set (1 km), prompted us to apply a tree-height filter only to samples with σ = 10 km.

### Quality Filtering

**FIGURE 1:**
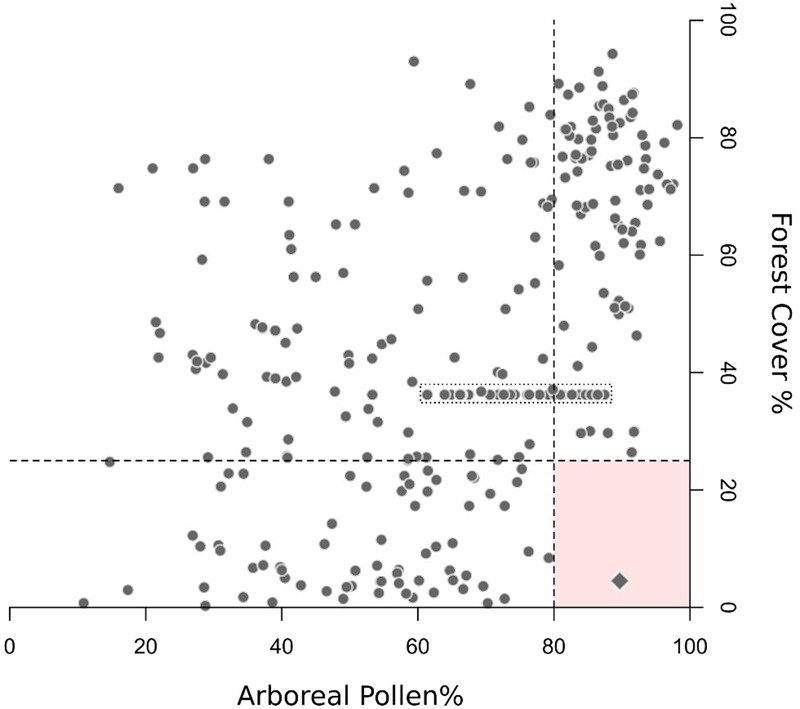
Arboreal pollen vs. forest cover for selected EMPD samples (“high-reliability” samples: location error < 100 m and age < –50 BP). Samples collected from open or species-poor environments are not included (e.g., tundra, sparsely vegetated mountain areas). The dashed lines delineate the boundaries used to detect low quality samples (AP > 80% and forest cover < 25%, see section “Quality Filtering”). Diamond symbol: the only high-reliability sample exceeding both values, located in the Northern Urals (sample name: “Lapteva_a83,” longitude 59.08828333, latitude: 59.52495). Despite being labeled as “forest undefined,” the sample is located in a treeless area above the local timberline (confirmed by both forest cover and vegetation height data sets). Still, the Globcover 2009 data set identifies its location as “Open (15–40%) needleleaved deciduous or evergreen forest (>5 m).” Nearby pixels are defined as sparsely vegetated. This contradictory information might imply a chronological mismatch between forest cover and pollen data, or inaccurate sample description and Globcover classification (possibly due to ecotonal transitions in the area). In either case, this sample does not invalidate the boundary conditions set by this quality filter. The dotted box includes samples collected in different locations within the same small lake (EMPD names: Kolaczek_f1-32), but with identical geographical coordinates (hence the identical forest-cover values). This situation highlights the need for both accurate sample geolocation and sample-specific pollen source area estimates.

The available EMPD metadata were used extensively in conjunction with additional land cover data sets in order to identify pairing inaccuracies between pollen samples and the related vegetation data (e.g., due to location or chronology errors, see section “Sources of Uncertainty”) through multiple lines of evidence. After duplicate removal, samples with estimated geolocation errors higher than 100 m were excluded from further elaborations. An additional reliability check was performed by comparing the elevation of each sample as recorded in the EMPD with the elevation for the same latitude and longitude extracted from a high-resolution Digital Elevation Model. Control elevation values were obtained from Mapzen’s Elevation Service^2^, accessed through the R package “elevatr” (Hollister and Shah, 2017). Any difference greater than 200 m resulted in the exclusion of the sample. Samples from riverine or estuarine contexts were excluded too due to the likely presence of pollen floated over long distances. Samples were also filtered to remove those with low pollen counts, retaining only samples with a total sum of terrestrial taxa greater than 100 pollen grains. The Globcover 2009 data set (Arino et al., 2008) was used in addition to the already-mentioned tree height and forest-cover data sets. Samples collected in open landscapes (e.g., sampling context identified as “treeless vegetation,” “pasture”) but falling in forested Globcover classes and having simultaneously more than 15% forest cover (Globcover lower limit for open forests) and median tree height > 0 were excluded from the analog pool. Similarly, samples collected in forested environments (e.g., “open forest,” “closed forest”) but falling within open Globcover classes and having at the same time forest cover < 15% and median tree height = 0 were excluded from the training set too. The high-reliability samples (estimated geolocation error < 100 m and estimated age < -50 BP; **Figure 1**) in the filtered EMPD were then used to find potential inaccuracies in surface samples with insufficient metadata. Any sample with arboreal pollen > 80% associated to forest-cover values < 25% was considered as potentially carrying low-quality information and excluded from the model. Beside geolocation and chronological inaccuracies, this filter addresses also samples collected under isolated trees, or in small thickets not detectable by the forest-cover data set. These threshold values are drawn from **Figure 1** and find support in the available literature. As an example, in open or semi-open contexts such as alpine meadows or arid shrublands, AP values are in the range of 40–50% (e.g., Davies and Fall, 2001; Court-Picon et al., 2006). Even above the tree line, in contexts affected by long-distance pollen transport, rare is the case where AP percentages reach values above 80% (e.g., Cañellas-Boltà et al., 2009). Nonetheless, given the common occurrence of long-distance airborne AP in species-poor environments (e.g., Pardoe, 2014), samples collected in extensive treeless contexts (e.g., tundra, alpine grasslands, deserts) are not included in **Figure 1** and are not affected by this filter. A comparable filter was not applied to situations with low AP and high forest cover values due to difficulties in identifying equally distinctive boundaries. See the discussion for additional considerations on data selection.

The filtering process led to the removal of ∼52% of the EMPD content, resulting in 2,526 usable samples (Supplementary Figure S2). Of these, 211 samples were distance-weighted using a σ-value = 100 m, 1,894 samples using a σ-value = 500 m, and 421 samples using a σ-value = 10 km.

### Past Forest-Cover Reconstruction

Forest-cover reconstructions are based on the MAT (Overpeck et al., 1985; Guiot, 1990; Jackson and Williams, 2004). The basic assumption behind the MAT is that pollen samples sharing similar combinations of taxa originate from comparable plant assemblages. The vegetation parameters of a modern pollen sample can therefore be transferred to any fossil sample sharing a similar palynological composition. In order to define how different two samples are, the floristic assemblages of both the fossil and the training sample are reduced to a single coefficient of dissimilarity. Squared-chord distance was selected as the dissimilarity index of choice because of its ability to differentiate between vegetation types (Overpeck et al., 1985; Gavin et al., 2003). Analog computations were performed using the R package ‘rioja’ (Juggins, 2016). The optimal maximum number of analogs, *k* = 8, was determined via leave-one-out (LOO) cross-validation. Reconstructed forest-cover percentages were then recalculated as the weighted average of the forest-cover values of the best analogs whose chord distance did not exceed a threshold T. The value of T is not straightforward to define since it is data dependent (Davis et al., 2015). We opted for a value of *T* = 0.3, as it has proved sufficient to discriminate between major vegetation assemblages in Europe (Huntley, 1990).

The fossil dataset was divided into 49 timeslices ranging from 12,000 to 0 calibrated years BP (years before AD 1950; hereafter BP). Each timeslice covers a 250-year window with the exception of the most recent one, which is asymmetric as it cannot project into the future, and therefore covers an interval of 185 years centered on 0 BP.

### Mapping Procedure

The reconstructed forest covers from each pollen record (i.e., point estimates) within each timeslice have been interpolated onto a uniform spatial and temporal grid using a four-dimensional (longitude, latitude, elevation, and time) Thin Plate Spline algorithm (TPS) fitted to a Digital Elevation Model (five arc-minutes resolution) using the R package ‘fields’ (Furrer et al., 2013). A three-dimensional TPS (latitude, longitude, elevation) was applied to the 0 BP timeslice in order to limit skewed results due to its unbalanced sample distribution around the 0 BP mark. This follows the same procedure used in mapping Holocene climate and vegetation used by Davis et al. (2003), Collins et al. (2012), and Mauri et al. (2015).

The maps of forest cover also include changes in European coastlines and ice sheets throughout the Holocene following Mauri et al. (2015). Small inland bodies of water are ignored in the reconstructed maps. Areas with low site/sample density over several time-windows have been excluded from our reconstruction, a process that was also used to define the borders of the study region. The results for some marginal areas (i.e., western North Africa and the eastern portion of the study area) are included but are not addressed in the paper, which focuses on the data-rich area of Europe north of the Mediterranean and west of 44°E.

To provide a comparison with traditional approaches based on the interpretation of arboreal pollen (AP) percentages, we also applied the same interpolation procedure to AP values (sum of woody taxa percentages, excluding dwarf shrubs and vines) for every fossil timeslice. The complete series of 49 forest-cover maps and 49 AP maps is included in the Supplementary Materials (Supplementary Figures S4, S5). All maps were produced using GMT 4.5.15 (Wessel et al., 2013).

Forest-cover error estimates were calculated for each timeslice by interpolating the standard error of the analog-based reconstruction for every fossil sample. The standard error itself was calculated as the standard deviation of the *n* best analogs (after applying the chord threshold) divided by the square root of *n*. This first interpolated error estimate was then added to the standard error generated by the interpolation process itself (Furrer et al., 2013; Mauri et al., 2015). For computational reasons, error estimates are provided in gridded form at a resolution of 1° (Supplementary Figure S6). Additional errors deriving from non-quantifiable uncertainties within data sources (see section “Sources of Uncertainty”) or from methodological limits (e.g., use of PFTs, lack of distinction between species-specific pollen productivity/dispersal; see section “No-Analog Situations and the PFT Approach”) can’t be quantified with the available data or technical knowledge. Therefore, these error sources are not accounted for in the calculation of error estimates.

**FIGURE 2:**
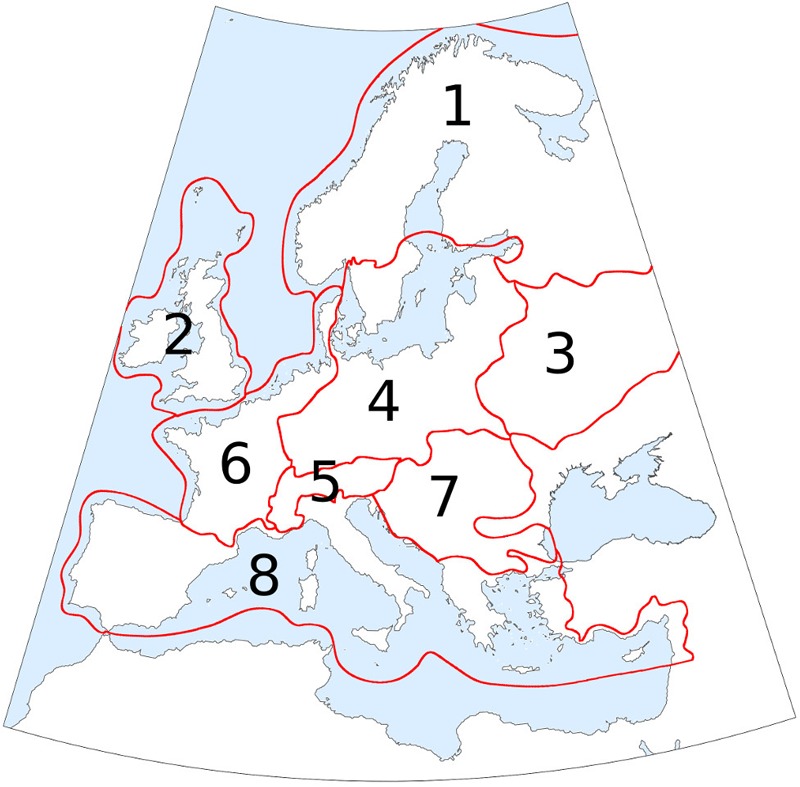
Study area divided into 8 regions to provide regional summaries of forest-cover changes. Names used in the text: 1. Boreal region; 2. British Isles; 3. Eastern European plain; 4. Central Europe; 5. Alpine region; 6. Atlantic region; 7. Dinaro-Carpathian region; 8. Mediterranean region. Region borders are loosely based on the European biogeographic areas proposed by Rivas-Martínez et al. (2004).

In order to provide regional summaries of forest cover and AP changes, the study area was divided into eight regions (**Figure 2**) and area-average estimates of forest cover were calculated for each region based on the gridded maps. The region borders are loosely based on the European biogeographic areas proposed by Rivas-Martínez et al. (2004). The area-average estimates of forest cover and AP percentages were then summarized in the form of time-series graphs covering the whole set of 49 time-slices.

### Evaluation of the Reconstruction Method

#### Performance of the Transfer Function and Interpolation Using Modern Data

The ability of MAT to reconstruct forest cover for the present time was first assessed using a two-fold cross-validation exercise. The modern pollen dataset was randomly split into two separate sets of equal size, subsequently using one as a training dataset to reconstruct the forest cover of the other and *vice-versa*. The whole process was repeated 999 times. The resulting *r*^2^, RMSEP, plus a simple reading of the residuals, were used to assess the basic performance of the method. The RMSEP has the same unit as the environmental variable, and is therefore expressed as percentages of forest cover.

An h-block cross-validation test (Telford and Birks, 2009) was not performed. With the h-block test, all samples within a distance *h* of a test sample are omitted from analog selection. This test is generally applied to climate reconstructions, in order to exclude or mitigate the effects of spatial autocorrelation in the training set. The European land cover is much less uniform than the European climate, being instead characterized by high fragmentation and spatial diversity even over short distances (Palmieri et al., 2011; Kallimanis and Koutsias, 2013). Due to the high spatial variability of pollen spectra/parent vegetation pairings, the h-block test might not represent an optimal solution for land cover reconstructions.

Additionally, we tested the joint ability of MAT and interpolation algorithms to reproduce the modern forest-cover patterns visible in Hansen et al. (2013). For this purpose, modern forest-cover values were reconstructed under less restrictive conditions via simple LOO cross validation. LOO-derived modern forest-cover values were then interpolated using the procedure described in Section “Mapping Procedure.” The same procedure was applied to AP percentages derived from the EMPD.

#### Reconstruction of Holocene Forest Cover in the Alps

The Alpine region provides an excellent context to test the performance of the 4-D interpolation due to its high topographic variability, the abundance of fossil pollen archives, and the numerous studies concerning local tree line and timberline dynamics. Therefore, as an additional test, we compared the MAT-based forest-cover values reconstructed at different elevations in the Alpine region with the data from Hansen et al. (2013) for the present and with independent macrofossil-based timberline/treeline reconstructions for the Holocene. To achieve this result, we grouped all the pixels (grid cells) falling within the Alpine region (n. 5 in **Figure 2**) into 200-vertical-meter elevation bands, except for those below 400 m which were grouped into a single band. This process was repeated for each of the 49 paleo-timeslices, as well as for the present day using both the LOO-based reconstruction and the map from Hansen et al. (2013).

As with the maps, areas with low site/sample density were excluded from the analysis. A lack of pollen sites at the very highest altitudes meant that for this test the analysis was limited to below 2,800 m throughout the Holocene, with a slightly lower limit of 2,400 m before 11,000 BP and 2,600 m from 10,000 to 11,000 BP when there were fewer sites above those altitudes.

A basic definition of “forest” in alpine contexts includes a minimum canopy cover above 40% (e.g., Burga and Perret, 2001) or 50–60% (Szerencsits, 2012). We adopt an intermediate threshold, following the upper altitudinal boundary of forest cover values >50% in our reconstruction. We interpret this minimum value as an indicator of widespread forested environments across the whole region, and use it as a simplistic threshold to track forest behavior in our reconstructions around the timberline ecotone (maximum vertical extent of alpine forests).

#### Comparison Between MAT and REVEALS Estimates of Past Forest Cover

Forest-cover reconstructions based on MAT have been evaluated against methodologically independent REVEALS reconstructions for selected fossil pollen records (five large lakes, >50 ha surface) in central and northern Europe. The REVEALS model (Sugita, 2007a) provides pollen-based estimates of regional plant abundances for 25 taxa (trees, shrubs, and herbs) in percentage cover and associated standard errors. Note that REVEALS estimates inferred from large lakes give the most reliable reconstructions of regional plant abundance (Trondman et al., 2015; Marquer et al., 2017).

We used five REVEALS target sites published in Marquer et al. (2014) for the method-independent evaluation of the MAT-based forest-cover reconstructions. These five pollen records were selected because the data was publicly available from EPD, and because the sites are all located in central and northern Europe, where pollen-productivity estimates are available to run REVEALS for the major pollen taxa (Broström et al., 2008; Mazier et al., 2012). For further details about the pollen records, see Marquer et al. (2014).

Several tests have been done to decrease the 500-year time intervals of the reconstructions in Marquer et al. (2014) to the lowest reliable time intervals: 400-, 300-, 200-, and 100-year time intervals were used for reconstructions and the results show that 200-year time intervals provide a good compromise between the size and number of the time intervals available and good REVEALS standard errors. The REVEALS model was therefore run for 200-year time windows covering the last 11,700 years: the number of time windows is dependent on the length of the record at each target site. The protocol for running REVEALS follows Marquer et al. (2014, 2017) and Trondman et al. (2015). For this test, the time resolution of the MAT estimates matches the REVEALS parameters.

The REVEALS estimates for the 25 taxa were grouped into open-land and forest categories, and the associated standard errors were calculated again. The REVEALS forest covers include covers of *Abies, Alnus, Betula, Carpinus, Corylus, Fagus, Fraxinus, Juniperus, Picea, Pinus, Quercus, Salix, Tilia*, and *Ulmus*, and represent the regional cover within 50 km around each target site.

## Results

### Model Validation

#### Performance of the Cross-Validation Exercise

**FIGURE 3:**
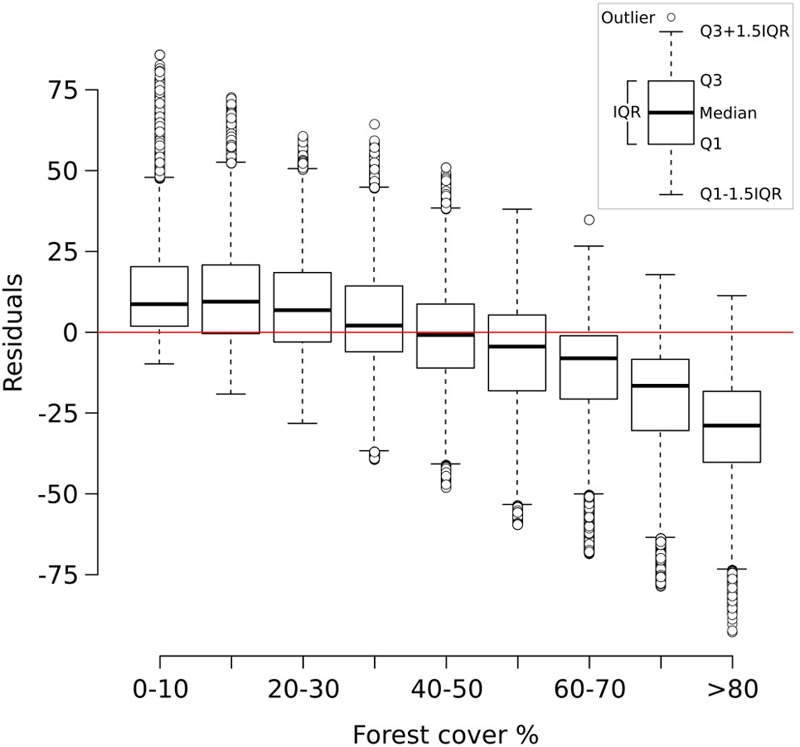
Box-and-whisker plot showing the residuals of the two-fold cross validation exercise (999 iterations) grouped into discrete forest-cover classes. Residuals are expressed as reconstructed minus observed forest-cover values. Q1 = first quartile; Q3 = third quartile; IQR = interquartile range.

The two-fold cross-validation exercise shows that the MAT-based forest-cover model is able to account for close to 50% of the variance (*r*^2^ = 0.49) and that the RMSEP (20.7%) is lower than the standard deviation of the data set (29%). These values reflect the wide array of uncertainties affecting the model (see sections “Sources of Uncertainty”, “No-analog Situations and the PFT Approach”, “forest-cover Underestimation”), yet they suggest that it can identify and reproduce a relationship between remote sensing data and pollen percentages. An analysis of the residuals of the two-fold cross-validation (**Figure 3**) highlights negligible bias in the 0–70% forest-cover range, with the median error lying well in the range of ±10% points. A larger discrepancy between observations and the reconstruction is especially visible at sites with very high forest cover (>80%), where the median reconstructed values tend to be more than 25% points lower than the observed forest cover. These differences might depend on a combination of factors. For instance, high-forest-cover samples (>80%) are the least represented in the calibration dataset, likely making them more prone to be affected by poor analogs. The lowest residuals are concentrated in southern Europe (Supplementary Figure S7) probably reflecting the limited representation in the modern dataset of large units of Mediterranean forest. These issues are revisited in the discussion section.

#### MAT–REVEALS Comparison

**FIGURE 4:**
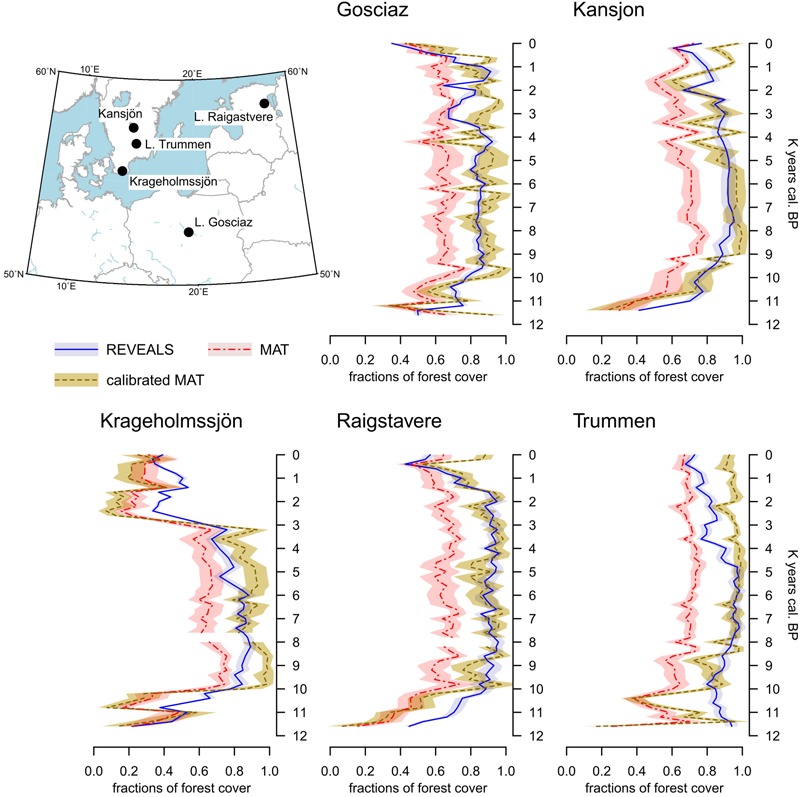
Location of five target sites and reconstructed forest-cover values based on MAT (calibrated and uncalibrated) and REVEALS estimates. The shaded area represents the standard error of each reconstruction. Calibrated values are obtained via the bias correction procedure detailed in Section “Bias Correction”.

**FIGURE 5:**
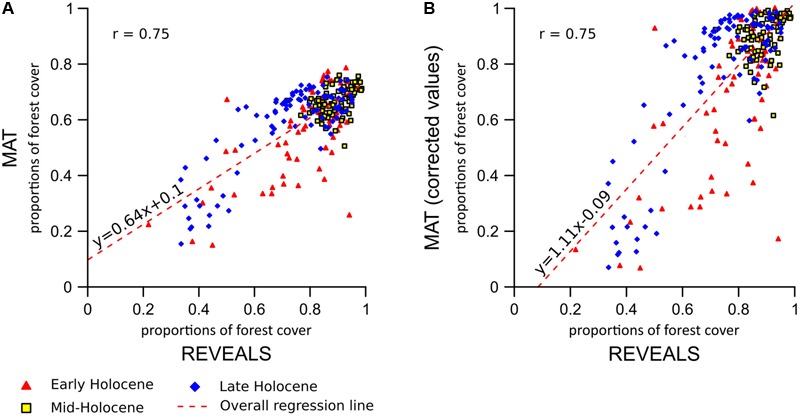
MAT–REVEALS comparison. **(A)** REVEALS vs. uncalibrated MAT values. **(B)** Reveals vs. calibrated MAT values. Overall statistical correlation values referred to **Figure 4**. Study time-window subdivision: Early Holocene: 11,700–8,100 BP. Mid-Holocene: 8,100–4,100 BP. Late Holocene: 4,100–0 BP.

The comparison between MAT and REVEALS estimates (**Figure 4**) shows generally similar trends (overall correlation coefficient: *r* = 0.75; **Figure 5A**). This level of agreement is notable, considering the quite different approaches of the two methodologies toward landscape reconstruction. Disagreements in absolute values constitute the main difference between the two models. The MAT assigns lower forest covers to early pioneering pine and birch woodlands (∼12,000–10,000 BP); during this phase, most of the closest modern analogs are chosen within the tundra biome or in proximity of the forest-tundra ecotone (Supplementary Figure S8). Differences between the models might then depend on a combination of factors, including potential forest-cover overestimation by REVEALS in Early Holocene contexts, and tree-height detection limits for MAT estimates. Major differences in terms of absolute values persist across all pairs after 10,000 BP, with the full development of mixed deciduous woodlands. The REVEALS curves rise to values higher than 85–90% before acquiring an overall stable neutral trend. After a comparable initial positive trend, the MAT curves stabilize at around 60–70%. Differences of ∼18–25 percentage points persist across the whole Mid-Holocene (Supplementary Table S4). This consistent behavior reinforces the results of the cross-validation exercise (**Figure 3**), pointing to a limited performance of the MAT in densely forested contexts. As noted in section “Performance of the Cross-validation Exercises,” the reasons behind this behavior might depend on a lack of suitable analogs in the training data set, in turn resulting from the low residual forest cover across present-day Europe. Different approaches toward forest-cover quantification might play a notable role too, i.e., sum of all forest-forming taxa, regardless of their habitus (REVEALS) vs. tree-height detections limits of the underlying forest cover map (MAT). Undetectable trees lower than 5 m might then account for at least part of the difference between the MAT and REVEALS curves.

Consistently, the only two no-analog situations (gaps in the MAT curves of Krageholmssjön and Raigstavere) are recorded at around 8,000 BP, testifying further to limits in the training data set concerning the reconstruction of early Mid-Holocene forests. The occurrence of no-analog situations in our reconstructions is addressed in section “Quantification of No-analog Occurrences.”

The agreement between MAT and REVEALS curves improves in the Late Holocene, when forest-cover decline brings the REVEALS values below 80%. The average difference in absolute values between the two reconstructions is reduced to ∼10–17 percentage points in the last 4,000 years of the sequences (Supplementary Table S4).

#### Quantification of No-Analog Occurrences

**FIGURE 6:**
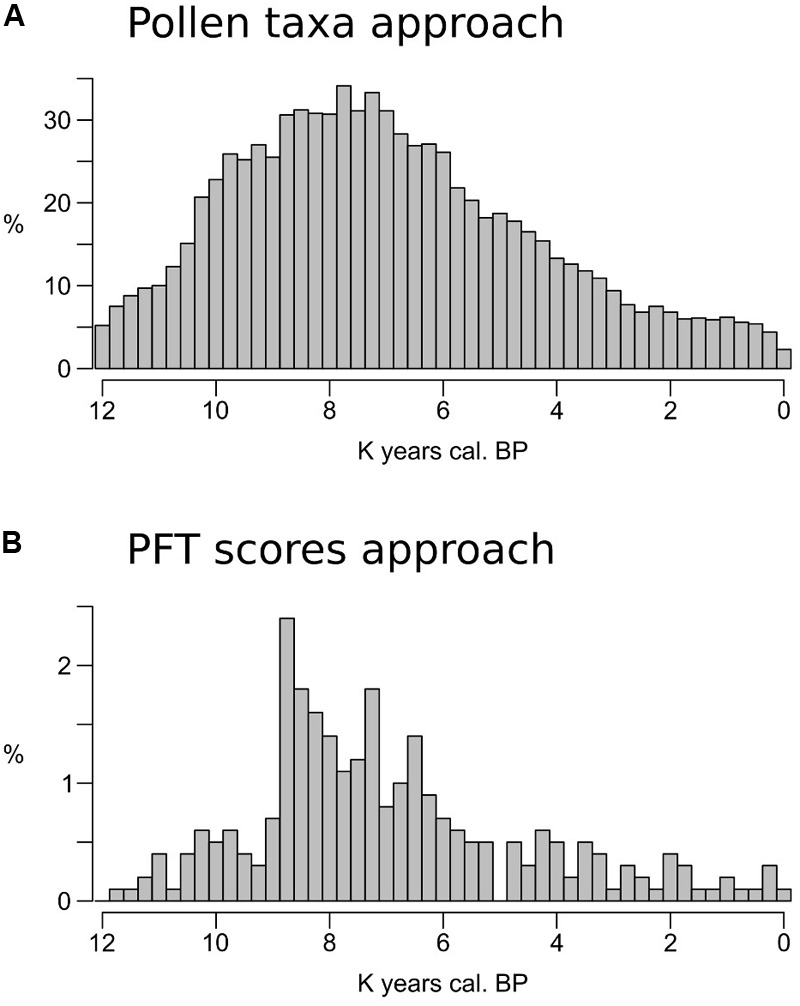
**(A,B)** Percentage of fossil samples with no close analogs in the training data set calculated for each time slice.

The MAT–REVEALS comparison shows few gaps in the MAT-based curves (sites of Krageholmssjön and Raigstavere, **Figure 4**), highlighting the occurrence of missing analogs and prompting us to explore the extent of this issue. For an appropriate comparison, we ran the MAT algorithm for the whole fossil and modern data sets using selected pollen taxa (i.e., without any transformation into PFT scores) and PFT scores (as described in section “Fossil and Modern Pollen Data”) separately. The full list of taxa used in this simulation is presented in Supplementary Table S5. The occurrence of no-analog samples for every timeslice is presented in **Figure 6**. Consistently with the parameters of our model, a no-analog situation is detected when the closest analog to a fossil sample has a squared chord distance higher than 0.3 (Huntley, 1990).

The taxa-based approach results in large portions of the fossil data sets presenting no-analog situations (**Figure 6A**). The period between ∼9,000 and 6,000 BP shows the highest values, with over 30% of the samples in each timeslice having no close analogs in the training set. The effect of the PFT approach is visible in **Figure 6B**: the highest no-analog percentages are still concentrated between ∼9,000 and 6,000 BP, but with values rarely exceeding 2%. A more detailed region-by-region subdivision is presented in Supplementary Figure S9.

#### Bias Correction

**FIGURE 7:**
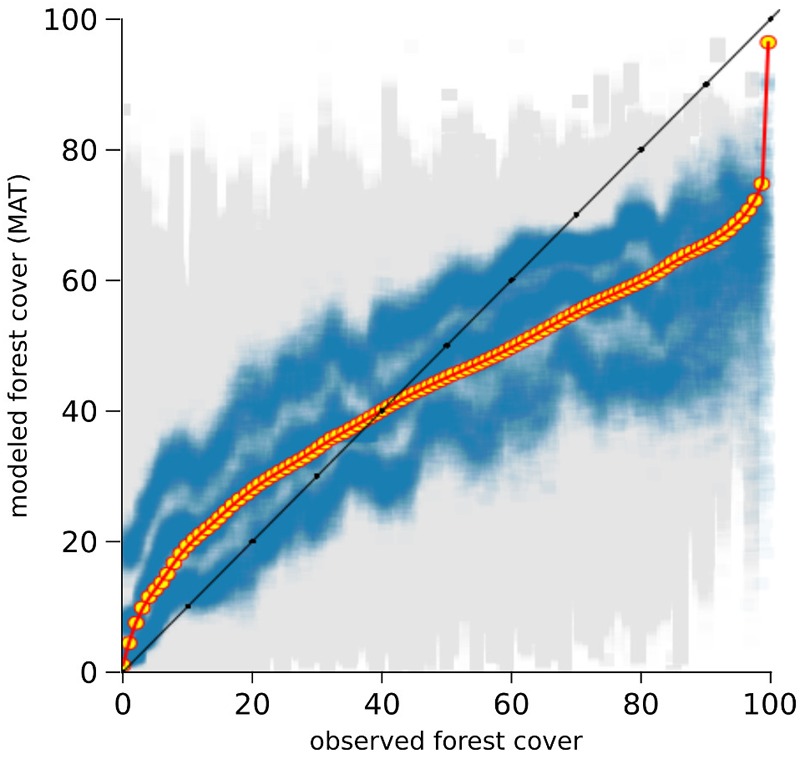
Calibration curve used to correct the modeled forest-cover values. Gray squares: modeled vs. observed forest-cover values from the two-fold cross-validation exercise. Blue squares: resampled first, second, and third quartiles. Yellow circles: modeled vs. fitted empirical quantiles. Red line: regression curve used for calibration of the modeled values.

**FIGURE 8:**
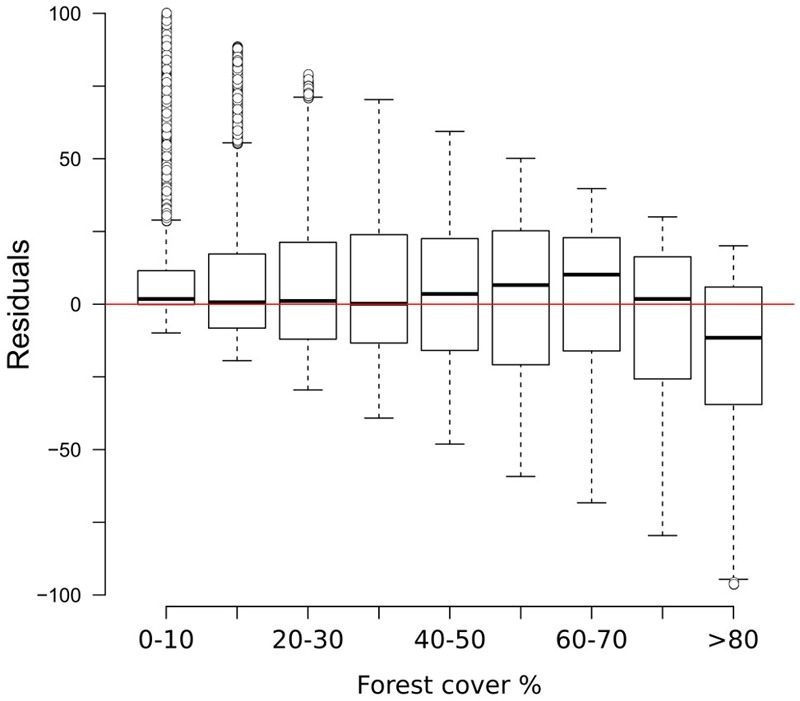
Box-and-whisker plot showing the residuals of the two-fold cross validation exercise (**Figure 3**) after the application of the calibration curve.

The results of the two-fold cross-validation exercise highlight a mild overestimation of forest cover at values ≲ 30% and, above all, a growing bias for values ≳ 50%. This systematic underrepresentation of high forest-cover values is clearly visible in the MAT–REVEALS downcore comparison, possibly suggesting a comparable bias spread across all Holocene reconstructions. Considering the seemingly systematic occurrence of this modeling error, we employ an empirical distribution correction approach (Lafon et al., 2013) to rectify the forest-cover reconstructions in our model. The results of the two-fold cross-validation exercise (999 iterations) were resampled using randomly distributed 5%-wide windows (10 randomly placed windows for every iteration) in order to address the predominance of low forest-cover samples in the training set. The first, second, and third quartile were extracted within each window. Robust empirical quantiles for modeled vs. observed forest cover were estimated via Quantile Mapping (R package ‘qmap,’ Gudmundsson, 2016), based on every second percentile between 1 and 100. A smooth spline regression curve was fitted to the resulting quantile–quantile plot (**Figure 7**) and used as a calibration curve for the modeled forest-cover values. The resampling strategy and regression parameters were tested via trial-and-error, opting for a solution that minimizes the bias for high forest-cover classes. The application of the bias correction curve to the two-fold cross-validation iterations is visible in **Figure 8**, where it shows a largely corrected median bias across all forest-cover classes. The correction of MAT values in the MAT–REVEALS comparison (**Figure 4**) shows how the calibration curve succeeds in closing the gaps between the two models during the Mid-Holocene (Supplementary Table S4), while at the same time preserving the relative trend between timeslices. It should still be noted that the bias-correction procedure is applied mechanically to all samples regardless of their age, composition or location. As a consequence, this simple calibration approach might result in an over- or under-correction of forest-cover values. Any decrease in agreement between MAT and REVEALS might be ascribed to these effects. Examples include the exacerbation of low forest-cover values in the Late Holocene section of Krageholmssjön, or marked shifts from 60–70% (uncalibrated) to >90% (calibrated) forest-cover values within the most recent timeslices of Kansjon and Raigstavere. Similarly, the monotonic behavior that characterizes most of the MAT-based Trummen curve results in a growing discrepancy between MAT and REVEALS from ∼5,000 BP onward. These differences are nonetheless counterbalanced by a general and notable improvement in the correlation between MAT and REVEALS (overall regression line with slope = 1.11 and intercept = -0.09; **Figure 5B**).

The forest-cover reconstructions presented in the following sections are based on the application of this bias correction procedure.

#### Evaluation of the Interpolation Procedure

**FIGURE 9:**
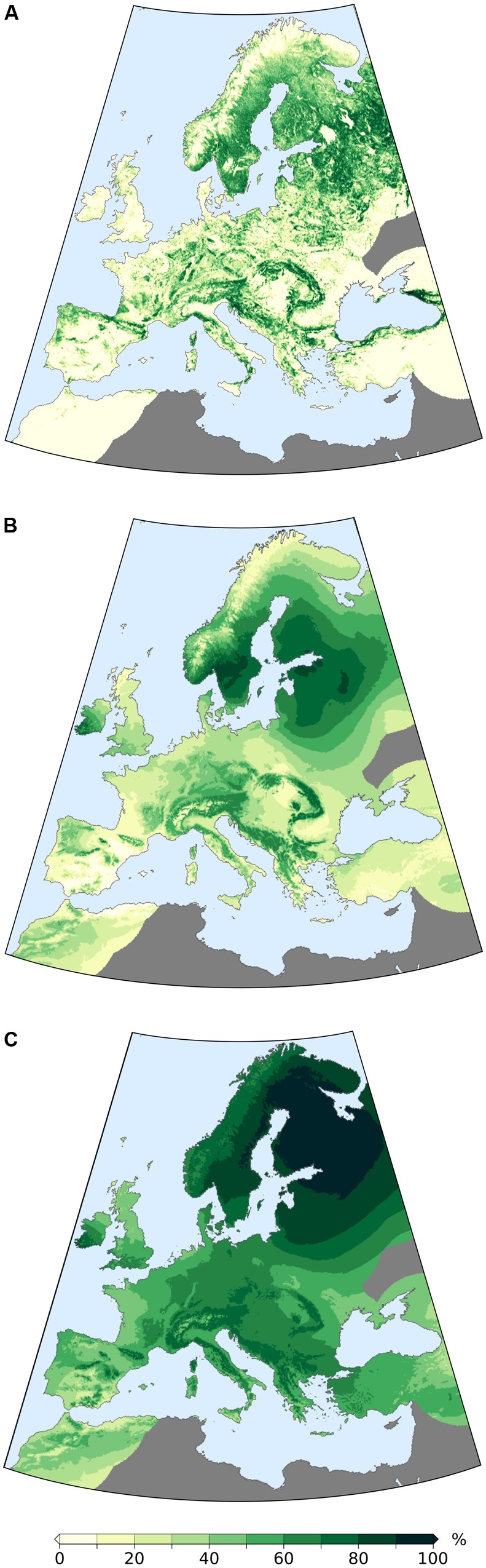
**(A)** Map of the study area with present day forest cover from Hansen et al., 2013; **(B)** Present-day forest cover reconstructed via MAT (LOO cross validation, calibrated) and 3D interpolation; **(C)** Interpolated present day arboreal pollen (AP) percentages from the EMPD and 3D interpolation. Dark gray areas are excluded from the analysis due to low site density.

**FIGURE 10:**
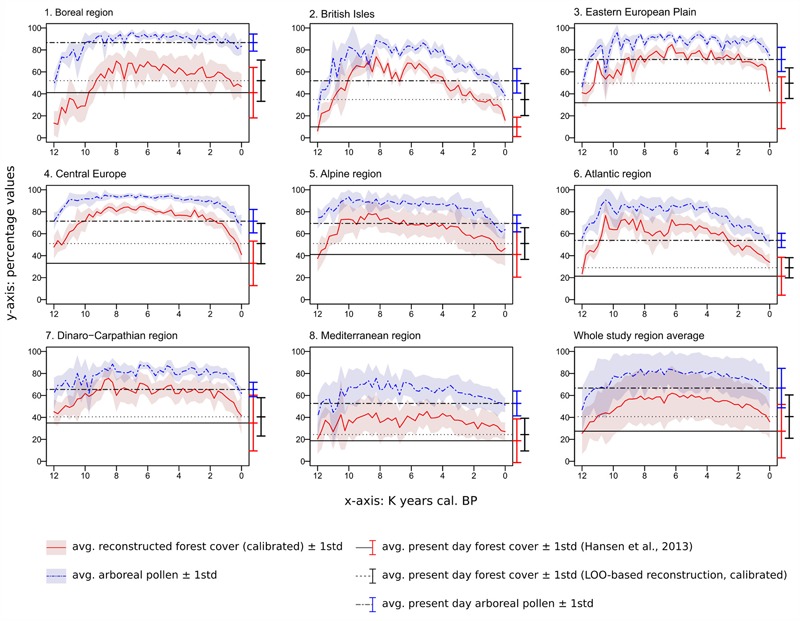
Time series for the whole study area and the eight regions (time vs. percentages of reconstructed forest cover and percentage of arboreal pollen). Areas covered by water or ice are not included in the calculation of average forest-cover/AP values and their relative standard deviation. The deviation from the mean does not represent the error of the reconstruction but is simply a measure of inter-regional variability. The horizontal lines provide a comparison with present-day values for each region, i.e., modern forest cover from Hansen et al. (2013) (**Figure 9A**), interpolated LOO-based modern forest cover (**Figure 9B**) and interpolated percentage of arboreal pollen (**Figure 9C**).

A visual comparison between actual modern cover from Hansen et al. (2013) (**Figure 9A**) and the MAT-based, interpolated modern forest-cover map (obtained via LOO method, **Figure 9B**) is used to evaluate the ability of the interpolation procedure to reproduce the main patterns in forest cover at the European scale. Higher forest densities are correctly reconstructed in mountain areas, notably at mid-low elevations in the Alps and along the Apennines, in the Balkan Peninsula and in the Carpathians, and partly in northern Iberia. In northern Europe, extensive forested areas are reconstructed around the Gulf of Bothnia and in western Russia. By comparison, the interpolated AP map displays similar distributions in central and southeastern Europe, but differs substantially in the remaining areas. Particularly visible is the pattern in northern Europe, where high AP values are present well beyond the northernmost limit of densely forested areas and show a generally limited spatial variability. A region-by-region comparison is presented in **Figure 10**, together with the full extent of the fossil-database reconstructions. The synthesis in **Figure 10** highlights how a combination of MAT and interpolation succeeds in producing forest-cover values that are within the range derived from Hansen et al. (2013), while AP values regularly exceed it. Non-overlapping values are produced only for the British Isles, presumably due to the relatively poor spatial distribution of modern samples available for this specific region (Supplementary Figure S2).

#### Forest-Cover Elevation in the Alpine Environment

**FIGURE 11:**
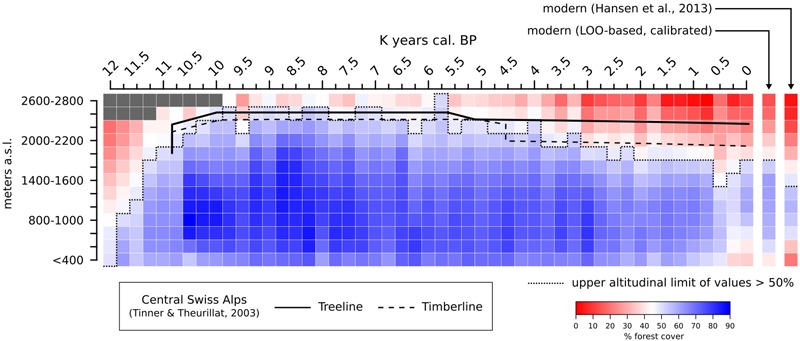
MAT-based forest cover values (calibrated) for the end of the Younger Dryas and the Holocene in the Alpine region grouped in discrete elevation classes. forest-cover data from Hansen et al. (2013) and LOO-based from the same region (derived from **Figure 9**) are added as a modern reference. Gray colored pixels and pixels above 2,800 m are masked due to lack of local pollen archives. Holocene macrofossil-based treeline and timberline curves from the central Swiss Alps are juxtaposed for comparison (digitized from Tinner and Theurillat, 2003).

A summary of the average MAT-derived forest cover in the Alps at different altitudes during the Holocene is presented in **Figure 11**. The overall lowest forest-cover values are reconstructed at the end of the Younger Dryas period, before 11,700 BP, when values > 50% are not recorded in the regional synthesis. A sharp increase in forest-cover percentages is immediately visible after 11,500 BP, rapidly leading to the maximum altitudinal development of dense Alpine forests in our reconstruction (∼10,000–7,000 BP). A gradual negative trend is then recorded between ∼7,000 and 2,250 BP, lowering the 50% threshold from ∼2,600 m to ∼1,800 m. Eventually, after ∼500 BP, values below 800 m decrease noticeably too, bringing the overall forest-cover situation close to the modern one across all elevation bands. In **Figure 11**, the 50% boundary in both the 0 BP timeslice and LOO-based modern timeslice is located at 1,800 m. By comparison, the satellite-based modern forest cover is placed 400 m lower, at 1,400 m. It should be noted that the satellite-based 1,400–1,600-m elevation class has a forest cover value only slightly lower than the threshold value (48.9%), causing a seemingly abrupt difference in elevation between the satellite-based and MAT-based forest covers. Comparing the 0 BP timeslice with the satellite-based and the LOO-based forest covers returns a correlation coefficient *r* = 0.98 in both cases. Similarly, comparing the satellite-based and LOO data results in *r* = 0.97.

### Forest-Cover Reconstructions

#### Late Pleistocene and Holocene Onset (**Figures 10, 12A,B**)

**FIGURE 12:**
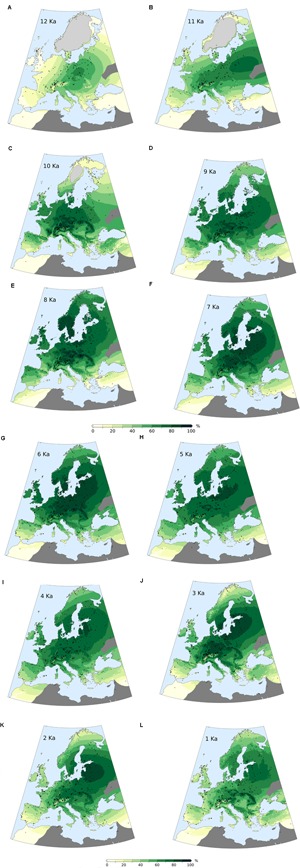
MAT-based forest-cover values (calibrated) for selected time slices during the past 12,000 years **(A–L)**. Gray crosses represent pollen sites locations. Light gray areas over northern Europe and Scotland represent Early Holocene ice cover. Dark gray areas are excluded from the analysis due to low site density.

During the last stage of the Pleistocene, the average forest-cover ranges from ∼6% (British Isles) to ∼48% (Central Europe), with an overall study area average of ∼25%. The highest percentages are reconstructed along a diagonal belt spanning from the circum-alpine area to central-eastern Europe (**Figure 12A**). The beginning of the Holocene is marked by a general increase in forest cover, with particularly steep trajectories in the British Isles, in the Atlantic region and, resulting in an isolated peak, in Eastern Europe.

#### First Half of the Holocene (**Figures 10, 12C–G**)

Maximum forest development is reached between ∼8,500 and 6,000 BP, with intensity and timing varying from area to area. Only the Atlantic region displays an early and isolated maximum around 10,500 BP. Wide standard deviations are visible across most regions within the same time slice. Positive forest-cover trends last until 6,250 BP in the Eastern European plain. The highest average values are recorded in Central Europe, with percentages often above 80%. The least forested region is the Mediterranean, where forest cover rarely exceeds 40%.

A phase of decline and stagnation lasting from ∼8,250 to 6,500 BP is visible in the British Isles, in the Atlantic and Dinaro-Carpathian regions, and in the Mediterranean. With the end of this phase, forest cover values recover and return approximately to pre-decline levels. A similar decline begins in the Alps after 8,250 BP, but is not followed by forest recovery.

#### Second Half of the Holocene (**Figures 10, 12H–L**)

The negative trend observed in the Alps since 8,250 BP continues during the second half of the Holocene. A comparable stable decline is visible across most regions generally from 6,000 BP. The contraction of forested areas varies across Europe in terms of intensity and timing, but invariably leads from Early/Mid-Holocene maxima to present-day values without any interposed major recovery phase. Only the Dinaro-Carpathian region shows a predominant neutral trend until ∼1,500 BP, then followed by a rather steep decline to present-day values. A notable increase in steepness occurs in Central Europe after 1,500 BP too, while in the British Isles, in the Easter European Plain and in the Alps occurs after ∼750 BP.

## Discussion

We reconstruct for the first time spatially continuous fields of forest cover over the entire European continent for the last 12,000 years based on quantitative criteria. The large spatial and long temporal scale of the study and the hundreds of individual sites on which it is based preclude a detailed analysis of individual local areas, so we restrict our discussion here to the main strengths and weaknesses of the model. It should be remembered that the reconstruction is based on aggregate results from several sites, and it is to be expected that individual site reconstructions may show different trends when viewed in isolation.

### Sources of Uncertainty

Williams and Jackson (2003), Jackson and Williams (2004), and Mauri et al. (2015) present an extensive summary of potential error sources affecting models based on pollen and modern analogs. These include the quantity and quality of the fossil and modern pollen data, chronological uncertainties, and the completeness and accuracy of attendant metadata. Whilst it is possible to apply rigorous quality control criteria, there is a direct trade-off in terms of the number of samples, and consequently the spatial, temporal, and ecological coverage. This can be particularly important for instance for the size of the modern pollen training set, and therefore the number of analogs that are available to the MAT. For instance, a sample count of 400–600 pollen grains belonging to terrestrial species is generally considered a safe minimum threshold to minimize statistical fluctuations of taxa percentages within a sample (Birks and Birks, 1980), with lower pollen counts being occasionally are occasionally imposed by the sample nature itself (e.g., bad preservation, low volume). Applying a minimum pollen count (terrestrial taxa only) of 600 grains would lead to a >60% sample loss in both the training and the fossil databases, affecting the spatial coverage and the general variability of both data sets. In our reconstruction we used a smaller sub-set of taxa selected from the total terrestrial count, and therefore we chose a smaller threshold of 100 terrestrial pollen grains as the minimum count.

The size of the count influences the taxonomic diversity of the sample, as does the skill of the analyst. Furthermore, additional vegetation parameters, such as habitus-related aspects are not always inferable from pollen data. Even when two species exhibit clearly different growth habits, their pollen grains might prove difficult to differentiate, as exemplified is exemplified by the *Betula* genus. Dwarf birch (*Betula nana*), an arctic/cool temperate low shrub, is not distinguishable from arboreal birches through grain size alone, requiring instead to calculate the ratio of equatorial diameter to pore height (Birks, 1968). It is not possible to ascertain to which extent this distinction constitutes a common practice among analysts (e.g., it is admittedly avoided in Solovieva et al., 2015). This specific issue is at least partly mitigated by the PFT approach: *B. nana* pollen grains are assigned to the arctic-alpine PFT, while undifferentiated *Betula* pollen may be categorized as either boreal summer-green *or* arctic-alpine depending on the remaining floristic assemblage of a given sample (Peyron et al., 1998 and Supplementary Tables S1–S3). Woody vegetation growth habit and size might be variously affected by environmental properties too, such as water availability and soil composition. These variables are not accounted for in the analog selection process, and can lead to associate fossil pollen spectra to incorrect modern vegetation parameters.

An additional source of uncertainty stems from the definition of forest in the Hansen et al. (2013) forest-cover dataset, which specifies that “forest” has a minimum height of 5 m. However, the LANDSAT sensors used to create the dataset do not detect tree height. Therefore, “forest” cover mapped in this dataset is subject to uncertainty and areas mapped as forest could in reality be something else, which would lead to irreconcilable differences between pollen spectra and tree cover. For example, forest could be mapped in the dataset when the vegetation cover is in fact rather a dense, tall shrubland, e.g., in the arctic. While indistinguishable to a satellite-based sensor, an arctic shrubland might have a very different floristic composition than, e.g., boreal forest, and this difference would be obvious in a pollen spectrum. This mismatch between observations from satellites and modern pollen samples would result in calibration uncertainty, where areas with similar forest cover have different pollen spectra, and vice versa.

Sample misplacement is another source for incorrect matches between pollen and satellite data. The high grid resolution of the remote sensing data and the small σ-value of the pixel-weighting equation result in a high sensitivity of the search window to shifts in its position. The EMPD provides reliability estimates for sample geolocation, which – combined with randomized location checks – have been used in the present work to exclude samples with known large uncertainties. This information is not available for the whole dataset, especially for older samples, due to gaps in the documentation or specific limitations (e.g., lack of GPS logging devices during fieldwork operations). Surface samples with unknown location reliability amount to >50% of the total. Despite this lack of metadata, they were included in the elaboration in order not to depopulate the analog pool.

An additional issue revolves around the availability of remote-sensing data. While satellite-based forest-cover data are available only for selected time periods, i.e., the year 2000 for Hansen et al. (2013) and years 1992–1993 in the case of DeFries et al. (2000), during the past decades European forest cover experienced a general increase, with localized areas of forest loss (Lambrechts et al., 2009; Potapov et al., 2015). For comparison, the EMPD samples with known age were collected mostly between the years 1978 and 2010. As a consequence, pollen spectra might be paired with non-contemporary vegetation patterns. The relevance of this issue can be proportional to the age difference between the two data sets, as a wider gap would simply leave more time for landscape transformations to take place. Nonetheless, chronology-based filters were explicitly avoided, since wide chronological differences do not necessarily imply significant vegetation changes.

All of these examples are meant to highlight how surface-pollen datasets would benefit from both enhanced sample density and metadata quality, ideally not only covering a wider range of vegetation assemblages, but also containing redundancy that would allow for stricter and task-oriented selection of samples. Furthermore, improvements in remotely sensed land-cover data would be desirable, e.g., using new sensors that directly integrate the spectral and physical characteristics of the vegetation into a single dataset. It is important to remember that good transfer function performance may equally be dependent on having a reliable calibration dataset as on having good pollen data.

### No-Analog Situations and the PFT Approach

The ability of MAT to find a proper set of analogs is challenged by the many factors influencing plant biology and distribution through time (Jackson and Williams, 2004). Pollen production and dispersal can be influenced by the age of the plant, with younger specimens producing less pollen than older ones (Jackson, 1994; Matthias et al., 2012). Similarly, varying concentrations of atmospheric CO_2_ have a direct effect on the production of flowering shoots, leading to an increase in pollen production with growing CO_2_ values (Ziska and Caulfield, 2000; Ladeau and Clark, 2006). Growth-speed limitations and interspecies competition might result in a lagged expansion within a potential bioclimatic space (e.g., Gardner and Willis, 1999). Furthermore, a growing human influence on the landscape in the second half of the Holocene played a role in the expansion of disturbance-dependent taxa outside of their niches (e.g., Perego et al., 2011), and possibly also contributed to early extinction events (Wick and Möhl, 2006). A combination of these and other factors led to the rise and decline of plant communities that potentially have no close parallels in contemporary vegetation.

Changes in pollen productivity within the same species represent an issue that is difficult to address and common to all pollen-based models. On the other hand, the sensitivity of the MAT to migration lags, extinction events, and gaps in the training data set can be lessened by avoiding a taxa-based approach. The use of PFT scores, as used in the present study, groups pollen taxa in broader classes defined by physical, phenological and climatic factors. The absence of specific taxa from an area does not hinder the performance of a PFT-based reconstruction as long as their potential bioclimatic space is occupied by species with comparable traits. The low occurrence of no-analog situations in our reconstructions (**Figure 6B**) proves the effectiveness of the PFT approach, and suggests that no-analogs do not affect the performance of the model. Yet a concentration of no-analogs between ∼9,000 and 6,000 BP remains notable. It occurs during the phase of maximum European forest development and highlights from a different perspective the noted model weakness with dense forest covers.

Still, the use of PFTs should not be regarded as a complete solution to the no-analog issue, but more as a viable and more robust alternative to taxa-based approaches. In fact, notable differences between taxa remain unaccounted for within the PFT method and contribute to the overall uncertainty of the model. As an example, beech (*Fagus*), lime (*Tilia*), and elm (*Ulmus*) share the same PFT (Cool-temperate summer-green; Peyron et al., 1998) despite having widely different pollen productivity estimates (beech: 0.76–6.7; lime: 1.3; elm: 0.8. Values are relative to the pollen productivity of Poaceae; Broström et al., 2008). Two samples with the same proportion of cool-temperate summer-green taxa (e.g., one composed primarily of beech and one of lime/elm) might then still be the product of different vegetation patterns. Beech spread gradually across Europe during the Holocene and is well represented in modern pollen diagrams (e.g., Magri, 2008). On the other hand, lime and elm are minor components of the present-day pollen landscape, having reached their palynological peak during the Early/Mid-Holocene (e.g., Huntley and Birks, 1983). Modern samples with beech might then be selected as best analogs for fossil samples lacking this taxon but containing lime and elm. The occurrence of such pairings – considering the potentially much higher pollen productivity of beech – could contribute too to the underestimation of Early/Mid-Holocene forest cover.

### Forest-Cover Underestimation

The results of the two-fold cross-validation exercise and the MAT–REVEALS comparison (see sections “Performance of the Cross-validation Exercise and MAT–REVEALS Comparison”) highlight a distinct issue with the reconstruction of high forest covers. A visible contribution to this issue is given by the limited availability of EMPD samples from highly forested contexts, but additional factors could play important roles too. Notably, the modern European landscape is largely deforested. As a consequence, any sample misplacement or incorrect distance-weighting could more likely lead to pair pollen samples with low forest-cover values. The use of PFTs could impact the model performance too, as noted in section “No-Analog Situations and the PFT Approach.” Most importantly, the MAT does not have any intrinsic ability to differentiate between local pollen sources and long-distance transport, an issue exacerbated by the generally poor pollen dispersal capabilities of open-landscape species (i.e., –simplifying –most herbaceous taxa). The under-representation of non-arboreal species results in samples with comparably high AP values but widely different forest-cover percentages (e.g., Broström et al., 1998 and **Figure 1** in the present paper), likely hindering a fine distinction between moderately and highly forested contexts.

These issues could be addressed by driving the analog selection process through a series of constraints. An example might involve calculating the average pollen productivity estimates of all PFTs, and reducing the analog pool to samples with comparable traits. Similarly, pollen accumulation rates could be used to detect predominant long-distance transport. Testing these solutions is currently prevented by a lack of necessary information, as pollen productivity estimates for the whole study area and for all taxa involved are currently not available. Similarly, the EMPD does not contain information concerning pollen accumulation rates. These limits led us to tackle the forest underestimation issue through a single correction equation applied to all sampling contexts and vegetation assemblages (see section“ Bias Correction”).

### Comparison With Current Land-Cover Change Narratives

Unraveling the fine reasons behind the regionally diverse forest cover trends remains beyond the scope of this paper. We will mostly constrain our analysis to a comparison between our data and the land cover dynamics emerging from qualitative interpretations and quantitative models. The interpolated results of our reconstruction draw a coherent picture of forest advancement and retreat spanning the last 12 millennia. While the limits of the method constitute an undeniable source of approximation, it is important to note that the reconstructions are often consistent with existing narratives of vegetation development from a regional to a continental scale, although these have virtually all been based on a qualitative interpretation of pollen data.

#### Late Pleistocene

The average forest cover across most of Europe just before the onset of Holocene warming points to a landscape dominated by non-arboreal vegetation. In agreement with this reconstruction, the traditional interpretation of pollen data describes a steppe-like environment with prevalent shrub and xerophytic vegetation covering most of the deglaciated areas of Western and Southern Europe (Walker and Lowe, 1990; Bakels, 1995; Guiter et al., 2005). Similarly, the presence of a steppic landscape matches several interpretations of Central Mediterranean (e.g., Bordon et al., 2009; Tinner et al., 2009; Combourieu-Nebout et al., 2013) and Eastern Mediterranean archives (e.g., Rossignol-Strick, 1995; Roberts et al., 2001; Langgut et al., 2011). In contrast, Lawson et al. (2004) point to a limited woodland contraction phase inserted in a context of gradual postglacial forest recovery. The expansion of an unforested environment during the Younger Dryas period is a phenomenon acknowledged in the Iberian Peninsula as well (Carrión et al., 2010). Here, the treeless conditions reconstructed in **Figure 12A** appear to be fitting for high-elevation sites and areas affected by a decrease in precipitation (Carrión and Van Geel, 1999; Carrión, 2002; Muñoz Sobrino et al., 2004), but might underestimate the presence of pine and oak woodlands growing in more favorable locations (Rubiales et al., 2010).

The higher forest-cover percentages emerging in central Europe probably reflect the presence of surviving Allerød pine/birch woodlands stretching from the Alpine piedmont (Wick, 2000) to Central Europe (Andres et al., 2001; Bos, 2001; Sobkowiak-Tabaka et al., 2017). Moving northward to the Baltic coast, the conditions inferred from pollen diagrams describe increasingly open birch and pine forests (Jahns, 2007; Nelle and Dörfler, 2008) gradually giving way to tundra-like conditions (Kihno et al., 2011). The relatively high forest cover values reconstructed around the Baltic Ice Lake may appear excessive considering the local importance of heliophilous taxa and the low pollen accumulation rates of forest species (Veski et al., 2012). Reworked pollen grains of thermophilous trees (e.g., Jahns, 2007) might arguably inflate model estimates. Nonetheless, macrofossil remains and pollen threshold values point to persistent populations of birch, pine, and spruce even at high latitudes and close to the ice margin (Kullman, 2002; Heikkilä et al., 2009).

#### Post-glacial Forest Recovery

The beginning of the Holocene is marked by warmer temperatures and greater moisture availability that promoted a rapid, wholesale transformation of land cover. The rise of forest-cover values over most of Europe is in line with the vegetation changes inferred from pollen diagrams. Existing woodlands densified and patchy tree stands replaced tundra parkland vegetation (e.g., Nelle and Dörfler, 2008; Robin et al., 2011). Growing forest density across Southern and Central Europe reflects the rapid expansion of thermophilous trees in pollen diagrams (Brewer et al., 2002; Giesecke et al., 2011). The 10,500 BP timeslice is characterized by an unusually high land-cover variability (high standard deviations in **Figure 10**), occurring together with visibly higher error estimates (Supplementary Figure S6). Significantly, this time window envelops the abrupt rise of hazel in central and southern European pollen diagrams (∼10,600 BP, Giesecke et al., 2011). This synchronous and fast paced event possibly resulted in widespread forest-cover differences at both intra-site (sudden palynological change between adjacent samples) and inter-site level (neighboring sites having different forest covers due to chronological factors – i.e., limits of their respective age–depth models), ultimately producing inflated error estimates propagating through site-poor areas. Peak levels of forest development are generally reconstructed between ∼8,500 and 6,000 BP, following the maximum extent reached by mixed deciduous forests (Brewer et al., 2002). Rising forest-cover values in Scandinavia reflect the colonizing wave of pioneering birch woodlands over the newly deglaciated landscapes (Bjune et al., 2004). Values higher than 50% are reconstructed in Northern Scandinavia after 8,000 BP, reflecting the widespread occurrence of birch and pine-birch forests at high latitudes (Seppa, 1998; Barnekow, 2000; Bjune et al., 2004).

Compared to other regions, there is only a modest increase in forest cover density in the Mediterranean between Late Pleistocene and Early and Mid-Holocene. These low values possibly reflect a combination of denser forests spreading inland and at upland sites, (Carrión et al., 2010; Sadori et al., 2011), and the contemporary development of coastal matorral/maquis shrublands (Carrión and Dupré, 1996; Pantaléon-Cano et al., 2003; Tinner et al., 2009; Calò et al., 2012).

#### Middle/Late Holocene Forest Cover Decline

During the second half of the Holocene, forest-cover percentages are characterized by negative trends that bring the curves to present-day values. Increasing human pressure is regarded as the primary driver behind such behavior (Kaplan et al., 2009; Davis et al., 2015; Fyfe et al., 2015), although the role of climate should not be dismissed (Marquer et al., 2017). forest cover over Northern Europe will have been influenced by the Early Holocene rise in temperatures and later Holocene cooling after the Mid-Holocene thermal optimum, whilst forests in Southern Europe were more influenced by similar trends in moisture (Davis et al., 2003). The estimated increase in carrying capacity of early agricultural societies implies an expansion of productive surfaces (croplands, pastures) at the expense of woodlands and wetlands. A growing exploitation of forest resources, primarily timber and fuel, must be accounted for as well, contributing to the general widespread and growing forest-cover reduction. The average picture described by our model is spatially and chronologically comparable to the synthesis presented by Fyfe et al. (2015), based on the categorization (Pseudobiomization, PBM) of Europe’s land surface into aggregated land-cover classes (e.g., forest, semi-open vegetation, open vegetation). At a pan-European scale, both models reproduce a period of maximum forest extent between ∼8,500 and 6,000 BP. Notably, this phase of relative stability is followed in both models by a very gradual forest decline. This negative trend then accelerates after 1,400 BP in Fyfe et al. (2015) and after 1,500 BP in our model (study area average, **Figure 10**). Additional similarities between the PBM and MAT can be found at a smaller scale too. Fyfe et al. (2015) describe a clear distinction between Western and Eastern Europe, with the first displaying consistently lower forest values. This East-West distinction is also visible in REVEALS estimates by Nielsen et al. (2012) and Trondman et al. (2015), and in the multi-model comparison by Pirzamanbein et al. (2014), confirming it as a robust feature of European land-cover history. The same pattern is clearly visible in our reconstruction since the Early Holocene, placing its origin before the expansion of Neolithic agriculture (Fyfe et al., 2015), and possibly reflecting – at least in part – an interplay of continentality and soil texture gradients (Nielsen et al., 2012).

The differences between Eastern and Western Europe are not limited to absolute land-cover values: the two regions show different forest-loss dynamics too. Fyfe et al. (2015) show a visible negative trend across their Western region (Western France) from ∼4,500 BP. On the other hand, Eastern Europe (Czech/Slovakia) presents only a very mild decline between ∼6,000 and 1,500 BP, then followed by a sudden and much sharper forest collapse. The Western France trajectory shares clear similarities with our Atlantic region curve, including a forest decline and recovery episode between ∼8,000 and 6,000 BP. The Czech/Slovakia situation matches forest-decline patterns in both Central Europe and in the Dinaro-Carpathian region (sudden decline after ∼1,500 BP) in the MAT-based model.

It is worth noting that quantitative landscape reconstructions by Nielsen et al. (2012), Pirzamanbein et al. (2014), and Trondman et al. (2015) agree on producing widespread Open Landscape values between ∼0 and 30% at 6,000 BP for central and northern Europe (i.e., ∼70–100% forest cover). These values are matched by the MAT-based reconstruction for the same area and time slice (range 60–90%), confirming further the validity of the MAT calibration algorithm.

In the British Isles, Woodbridge et al. (2014) set the onset of Neolithic land use in Britain at ∼6,000 BP; this is also when our MAT-based forest cover begins its negative trend. A notable difference consists in a re-afforestation event detected by Woodbridge et al. (2014) between 5,400 and 4,200 BP which temporarily interrupts the overall negative Late Holocene decline. This event is not visible in the MAT-based curve, possibly due to a non-perfect comparability between the PBM land cover classes and the continuous MAT reconstruction.

The Mediterranean region shows an overall weak and gradual increase in open areas when compared with other more dramatic regional dynamics. The largely open landscape reconstructed during the Early/Middle Holocene is likely to play a primary role in lessening any evidence of radical human impact, yet other factors might contribute to this modest change in land cover too. As an example, even severe disturbance events (e.g., Cremaschi et al., 2006) might simply not be visible due to the spatial and temporal coverage of the underlying fossil database.

#### Vertical Performance of the Interpolation Procedure in the Alpine Region

It has been widely argued that pollen percentages alone are not sufficient to infer the local presence of tree populations in extreme environments at the limits of tree growth (e.g., Ponel et al., 1992; Tinner et al., 1996; Ali et al., 2003). Under these conditions, flowering season is negatively affected and trees tend to propagate via vegetative multiplication and not through sexual reproduction (Black and Bliss, 1980; Kullman, 1992; Charalampopoulos et al., 2013), likely altering their palynological signature. The combined interpretation of pollen percentages and influx may help in identifying clearer vegetation thresholds (Hicks, 1994), potentially countering the effect of upslope and long distance pollen transport. Still, the presence of macrofossils is considered the most solid piece of evidence to reconstruct vegetation history across the timberline (e.g., Birks and Birks, 2000). Considering the limits of pollen data in high-elevation contexts, in the present section we evaluate the capabilities of our model to detect much broader forest dynamics. Importantly, it again should be remembered that we reconstruct the area-average forest cover of the entire Alpine region, and that this might variously differ from individual site records and smaller regional studies.

By end of the Younger Dryas period (11,700 BP), alpine forests became rather open even in the lowlands (Ammann et al., 2007; Vescovi et al., 2007). This situation appears to be broadly compatible with our model, which does not produce values >50% before 11,750 BP. The following abrupt positive shift of the 50% threshold matches well the upward movement of forests at the transition into the Holocene (Tinner and Kaltenrieder, 2005). In their simulation of alpine biomass, Heiri et al. (2006) detect a short-lived dieback event around 10,500 BP. Notably, the MAT-based model records a mild stagnation episode of the 50% threshold approximately around the same period, between ∼11,250 and 10,500 BP (**Figure 11**). Differences in the scale of the reconstructions, chronological uncertainties and temporal interpolation might explain the slight offset between the two events. Speculatively, the forest-cover stagnation seen in our model could be ascribed to the combined effects of the cool Preboreal oscillation (11,363/11,100 BP; Schwander et al., 2000) on alpine forests (Gobet et al., 2005), and to the dieback identified by Heiri et al. (2006). Major dieback events are described by Heiri et al. (2006) also between ∼9,500–9,300 BP and ∼6,500–6,000 BP, both matching mild – but not necessarily significant – elevation drops of the 50% threshold in our reconstruction. The prominent dieback described by Heiri et al. (2006) at 8,000 BP does not appear to be resolved in **Figure 11**, probably because of the temporal resolution of samples in the underlying pollen dataset. An overall regional change is anyway visible in **Figure 10**, where alpine forest-cover values drop noticeably after ∼8,250 BP. Between 9,750 and ∼7,000 BP the 50% threshold fluctuates mostly between 2,400 and 2,600 m. The lower value is in good agreement with the timberline elevation proposed by Tinner and Theurillat (2003) for the Swiss Central Alps, based on macrofossil analysis (**Figure 11**). Notably, estimates for maximum forest limits range from 2,500 m (Tessier et al., 1993) to 2,800 m (Talon, 2010) in the French Alps. After ∼7,000 BP, the upper limit of the 50% threshold begins to drop quite steadily, likely reflecting the contribution of different local forest dynamics to our regional average. Treeline decline is visible as early as 6,350 BP in the Austrian Alps (Nicolussi et al., 2005), while it is recorded after ∼5,750 in the Central Swiss Alps (**Figure 11** and Tinner and Theurillat, 2003). The abrupt timberline drop reconstructed by Tinner and Theurillat (2003) between 4,750 and 4,250 BP appears to be diluted in time in our reconstruction (**Figure 11**). The 50% threshold never exceeds 2,400 m after 5,750 BP, possibly reflecting the origin of anthropogenic pastures above 2,300 m during the Copper Age (∼5,600 BP, Pini et al., 2017) and a subsequent continuous and growing exploitation of the high alpine landscape.

Ultimately, a comparison between our model and independent palaeo-ecological studies suggests that a combination of MAT and interpolation can reproduce broad vertical vegetation patterns (i.e., fluctuations of the upper limit of region-wide dense forests) at a resolution sufficient for large-scale vegetation models. The reconstruction of sharper forest-vs.-no-forest cutoffs remains beyond the capability of the interpolation procedure. Finer results could be achieved through the incorporation of complementary proxy data (i.e., plant macro remains, charcoal), although with the caveat that such data remain much less widely available than pollen records.

## Conclusion

In this study we applied the MAT to generate a continuous, continental-scale reconstruction of European forest-cover spanning the last 12,000 years. Our reconstruction follows the general methodology published by Tarasov et al. (2007) and Williams et al. (2011). When compared with these studies, our work presents a series of improvements that extend the reliability and spatial/temporal coverage of the reconstruction. An increased number of samples in the modern database allows for stricter quality control and provides a better representation of different vegetation types. A larger fossil database allows for higher spatial density and better dating control. The satellite-based forest-cover data set used in previous studies (DeFries et al., 2000) was replaced with a higher resolution map (Hansen et al., 2013), leading to more reliable pairings between vegetation and modern pollen data. Furthermore, pollen taxa were aggregated into PFTs in order to reduce the occurrence of no-analog situations. The main improvement in mapping our results is the use of four-dimensional spatial interpolation (Mauri et al., 2015). This procedure extrapolates reconstructed vegetation data between a grid of fossil sites in both space and time, producing a set of forest-cover maps with continuous coverage and regular time-step at a continental scale. These characteristics represent a rather unique feature within the category of pollen-based quantitative vegetation reconstructions, and make these maps suitable for comparison with vegetation models and anthropogenic land-cover change scenarios.

The reliability of our reconstructions is supported by a comparison with methodologically independent data sets. forest-cover reconstructions based on the MAT (this study) and on the LRA were compared for selected sites (Marquer et al., 2014) in the circum-Baltic area. Both methodologies present widely comparable trends. Notable divergences are visible especially during the Mid-Holocene, with REVEALS consistently producing higher values. The limits of the MAT concerning the reconstruction of high forest covers can be ascribed to limitations within both the available training data set and the method itself. A visible role is played by the scarce representation of densely forested areas in the EMPD, an issue furtherly exacerbated by the averaging nature of the MAT. Furthermore, the MAT does not have any intrinsic capability to correct for differential pollen productivity, or identify samples largely affected by long-distance pollen transport. These problems could be addressed through targeted sampling campaigns, aimed at improving the coverage of specific vegetation patterns in the EMPD, and possibly by introducing constraints based on pollen productivity and accumulation rates. Given the systematic occurrence of biased MAT results and the current inapplicability of constraint-based procedures, we opted to tackle this issue through a statistical approach to bias correction. We used Quantile Mapping to extract a calibration curve from the output of the cross-validation exercise. The effectiveness of this calibration approach is testified by a visible reduction of the overall bias in both the cross-validation test and the comparison with REVEALS data.

The resulting continental-scale maps draw a coherent picture of vegetation development across Europe since the end of the Younger Dryas period. forest cover trends fit well in accepted narratives based on qualitative and quantitative interpretations of palaeovegetation data.

As a further test, we evaluated the vertical performance of the interpolation procedure against macrofossil-based tree line/timberline studies in the Alps. Detecting ecotones in alpine environments via pollen percentages is a task affected by wide margins of error. Consequently, we focused our comparison on broader vegetation patterns (i.e., variations in the upper limit of region-wide dense forests). A combination of MAT and spatio-temporal interpolation proved able to track major tree cover fluctuations in proximity of the timberline, thus reinforcing the broad reliability of the interpolation algorithm across a highly dynamic landscape.

## Author Contributions

MZ and BD designed the research. MZ performed the data analysis and presentation. LM contributed the independent (REVEALS) forest-cover reconstructions to aid method validation. MZ, BD, LM, SB, and JK were involved with substantial contributions in refining the methodology, writing, and reviewing the manuscript.

## Conflict of Interest Statement

The authors declare that the research was conducted in the absence of any commercial or financial relationships that could be construed as a potential conflict of interest.

## Footnotes

1 www.pangaea.de

2 www.mapzen.com

## Supplementary Material

The grid files of reconstructed forest cover, associated error estimates, and arboreal pollen percentages are available for download from https://doi.pangaea.de/10.1594/PANGAEA.886656
