# Supplementary material for: European Forest Cover During the Past 12,000 Years: A Palynological Reconstruction Based on Modern Analogs and Remote Sensing
Source: Front Plant Sci. 2018 Mar 8;9:253. doi: 10.3389/fpls.2018.00253 (PMC5852684; doi:10.3389/fpls.2018.00253)
Supplement: Supplementary file 1 [file Data_Sheet_1.pdf]

Supplementary Material

European Forest Cover During the Past 12 000 Years: A Palynological Reconstruction Based on Modern Analogues and Remote Sensing

Marco Zanon*, Basil. A. S. Davis, Laurent Marquer, Simon Brewer, Jed O. Kaplan

***Correspondence:**

Marco Zanon

mzanon@gshdl.uni-kiel.de

1 Supplementary Figures and Tables

1.1 Supplementary Figures

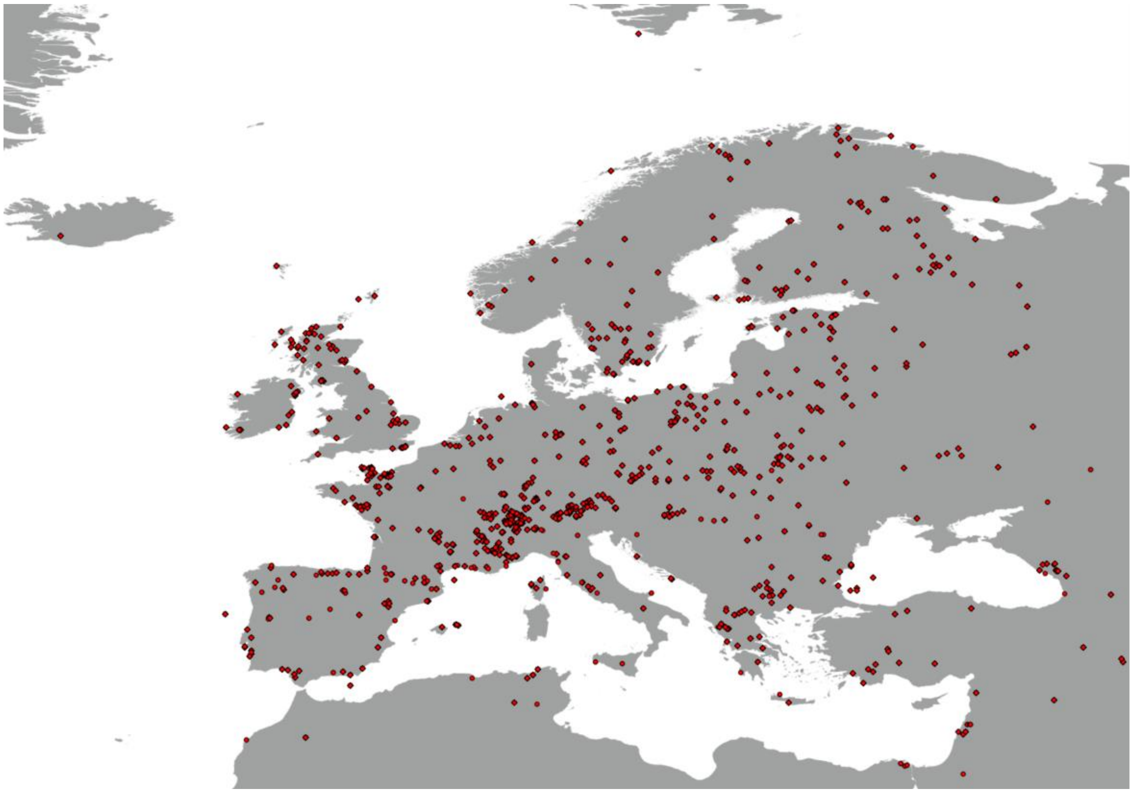

Figure S1. Distribution of fossil pollen sequences used in the present paper.

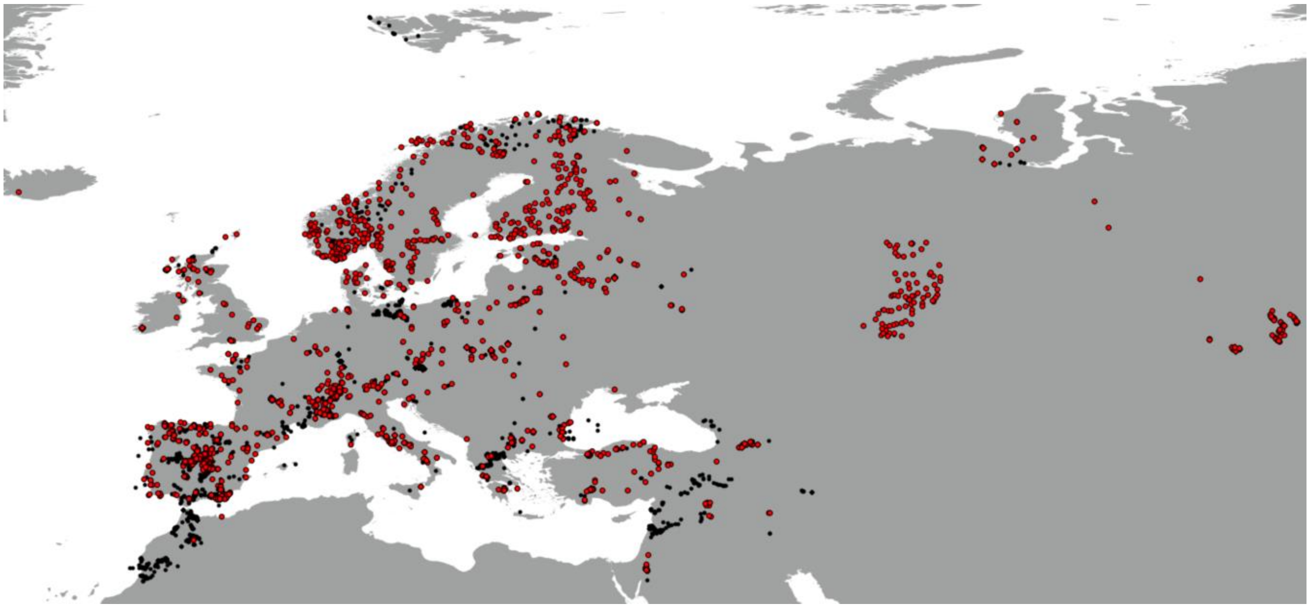

Figure S2. Distribution of surface samples in the European Modern Pollen Database (all dots). Red dots: extent of the database after quality filtering (see section Quality Filtering in the manuscript).

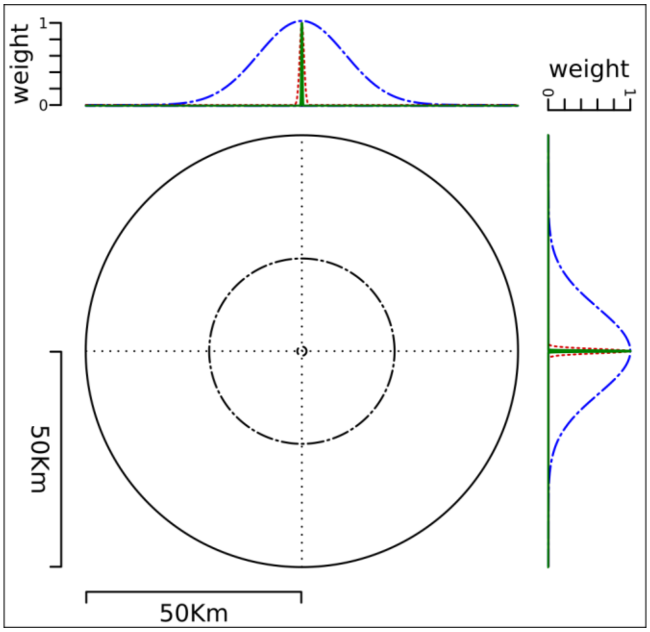

Figure S3. Exemplification of weight distribution using a 2d Gaussian function with a range of 50 Km (solid-line outer circle). Dash-dots blue line: σ =10km. Dotted red line: σ =500m. Solid green line: σ =100m. For reference, the inner dot-dash circle shows the limits of the 0.1 weight threshold for σ =10km (ca. 21.5Km radius). The small central dashed circle shows the same weight threshold for σ =500m (ca. 1km radius). During the extraction of forest cover values, all weights falling within each search radius were scaled to a sum of 1.

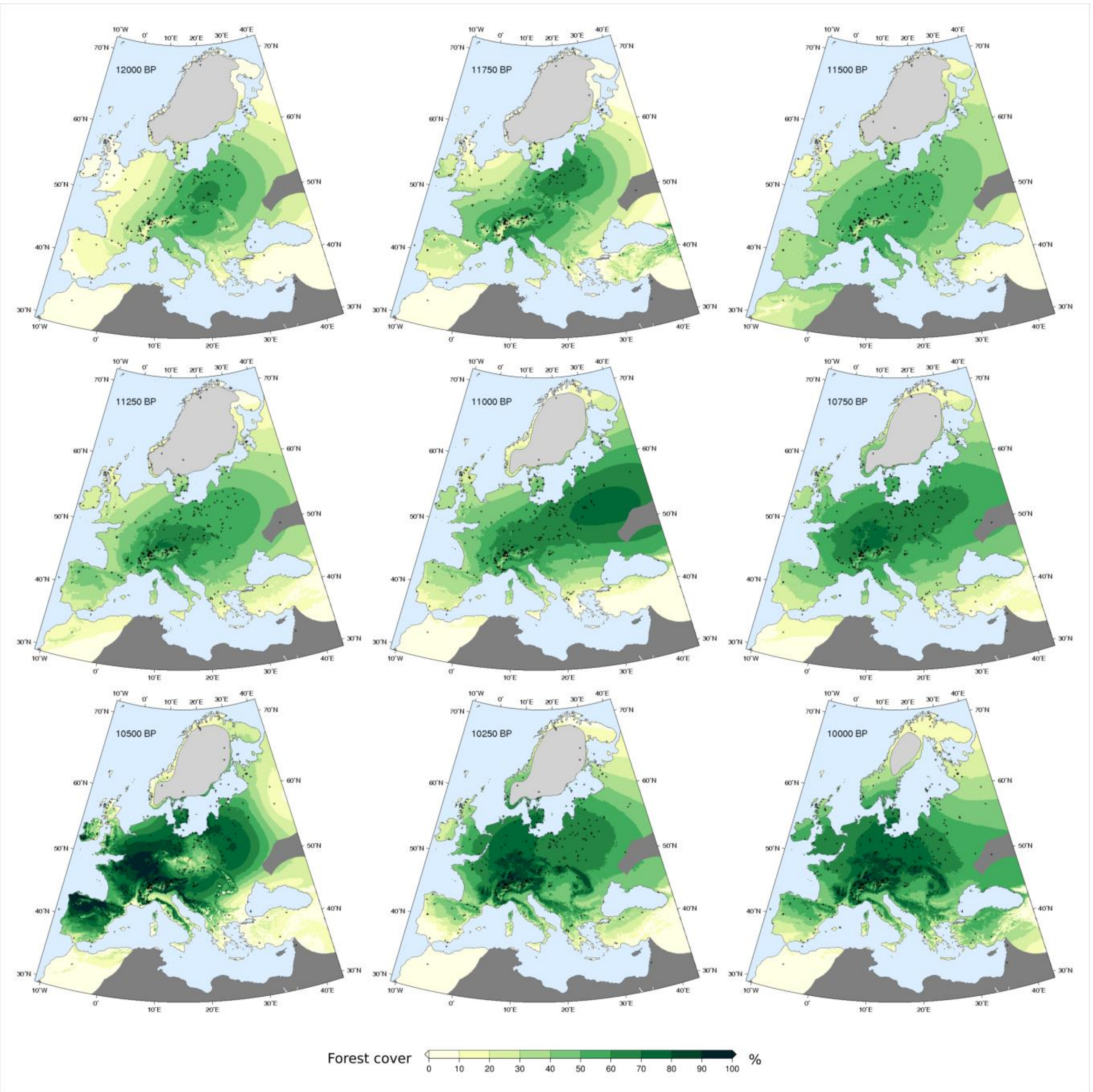

Figure S4. Holocene interpolated tree cover maps. Light grey areas over Scandinavia and Scotland represent Early Holocene ice cover. Dark grey areas are excluded from the analysis due to low site density. Grey crosses represent the location of pollen sites used for the interpolation.

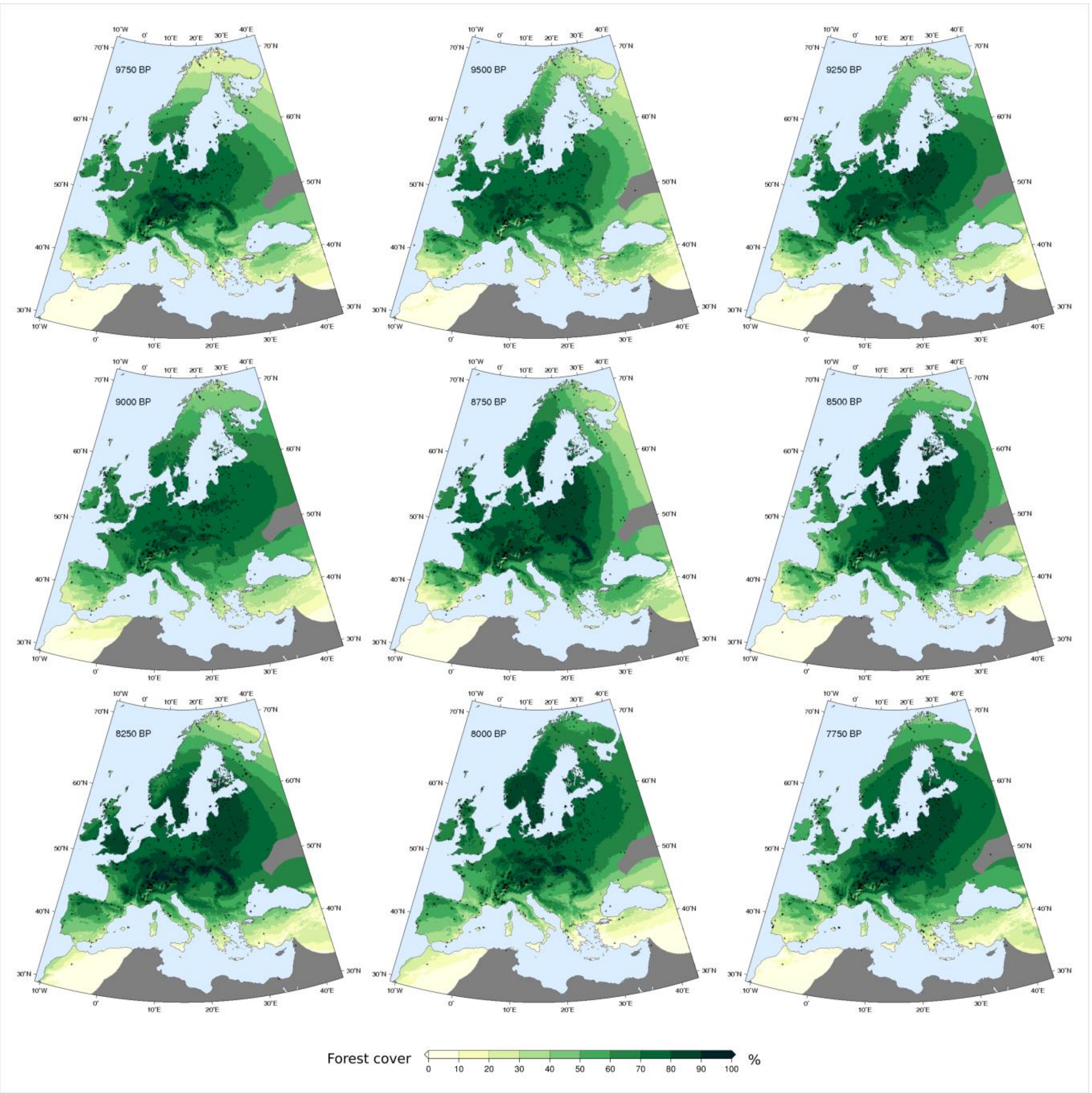

(continues from fig. S4)

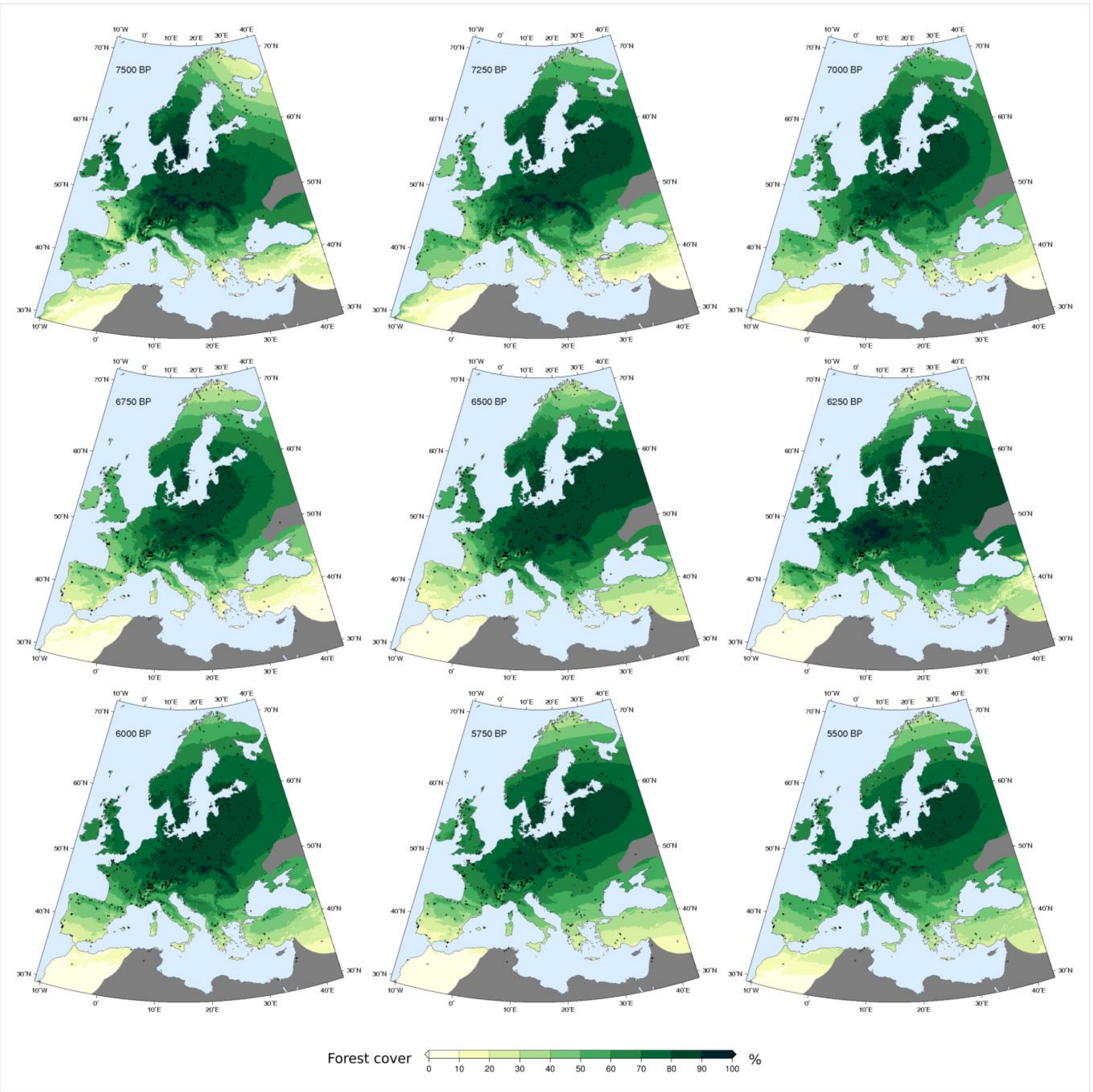

(continues from fig. S4)

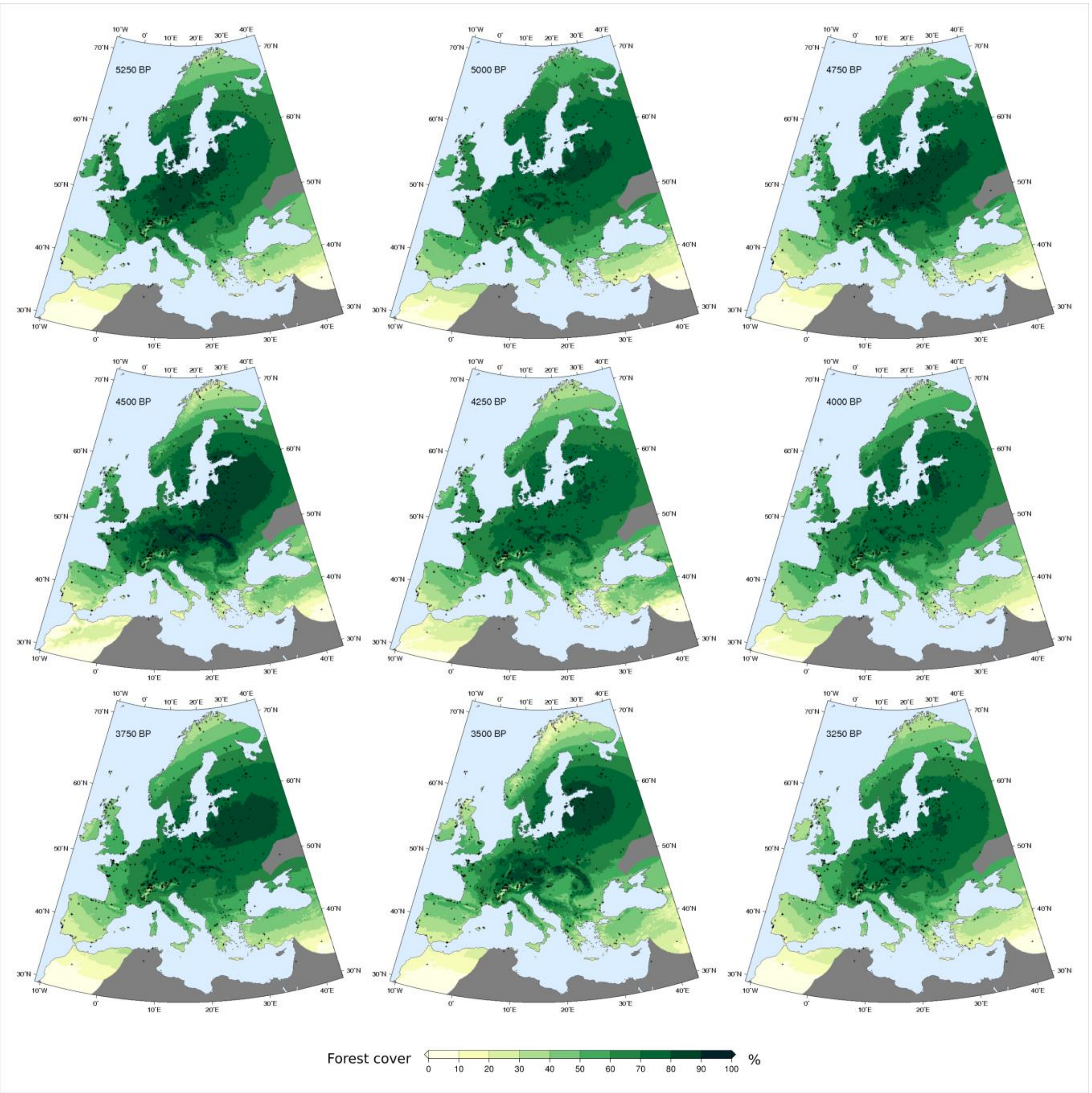

(continues from fig. S4)

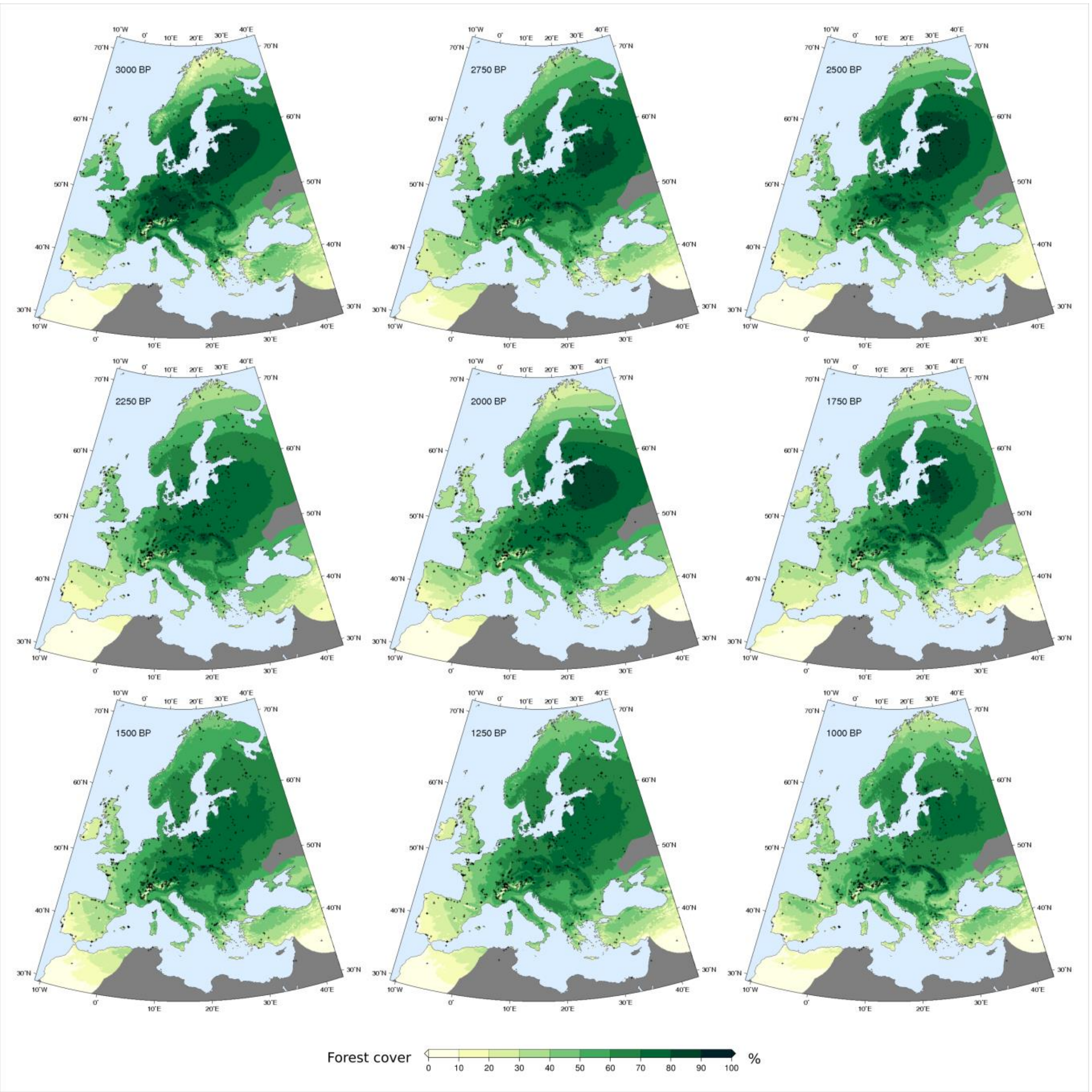

(continues from fig. S4)

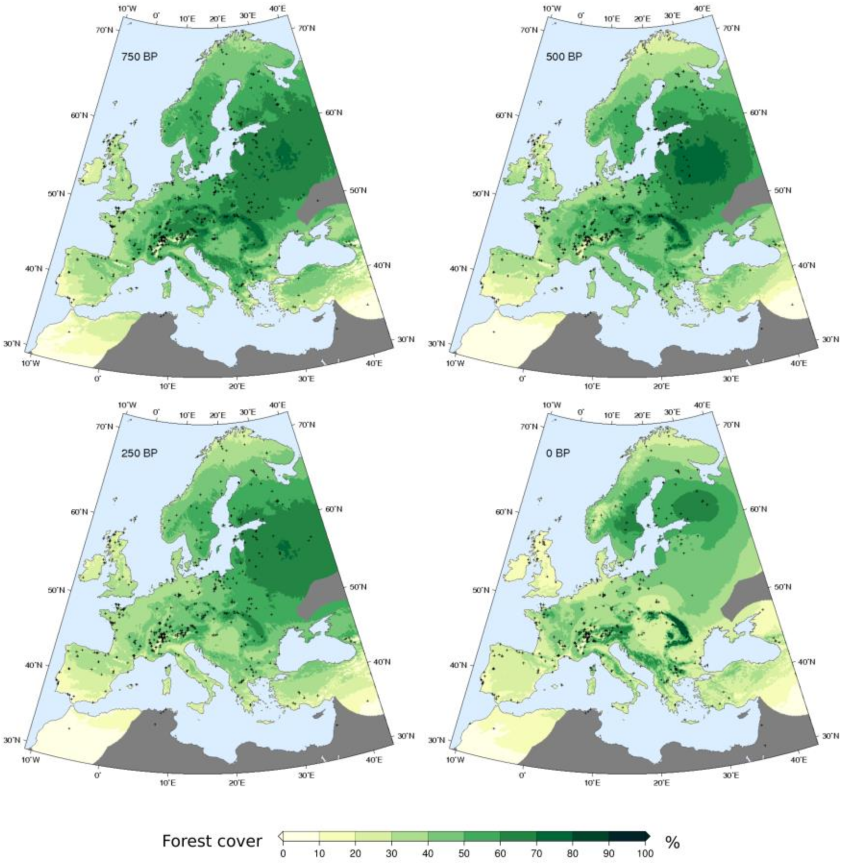

(continues from fig. S4)

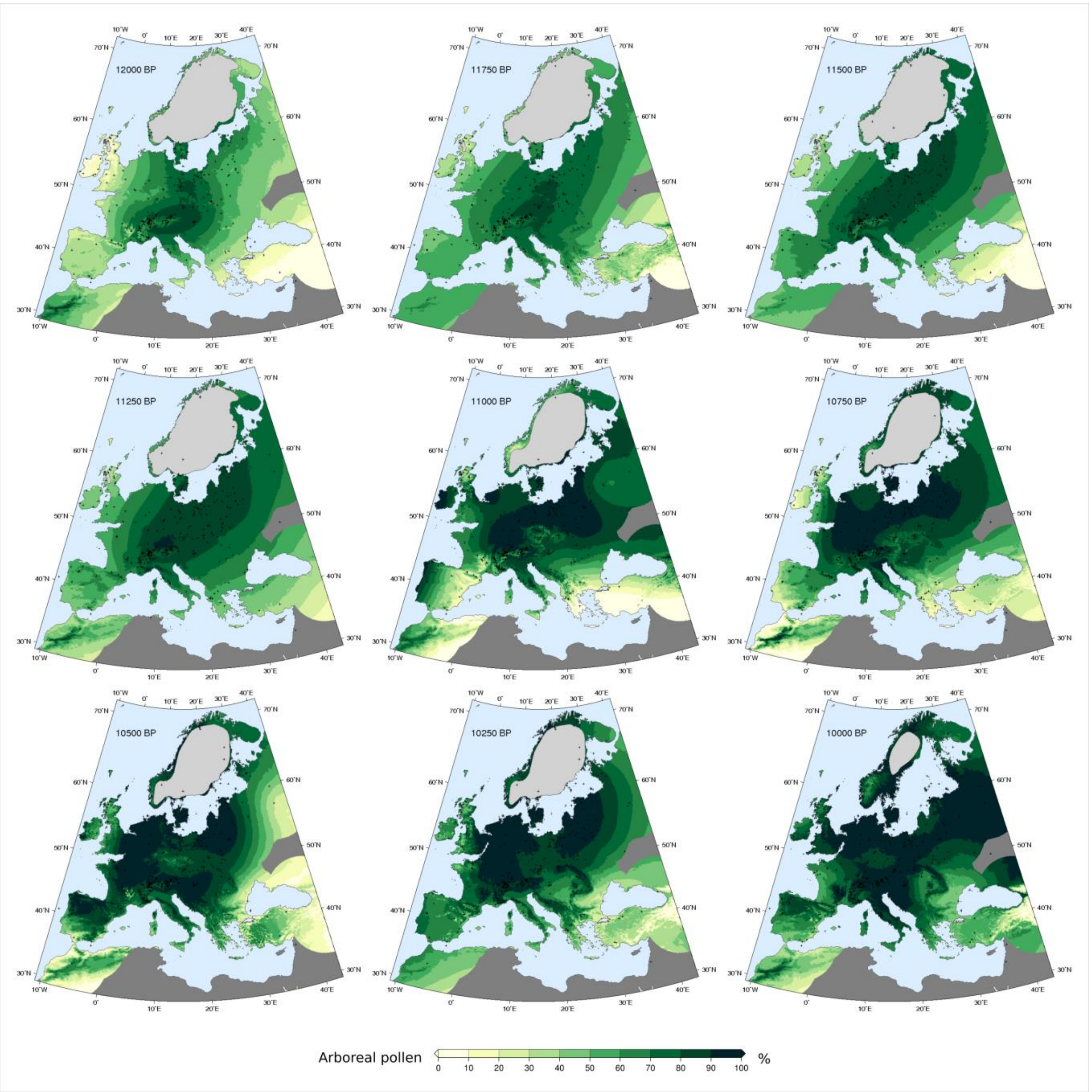

Figure S5. Holocene interpolated arboreal pollen maps. Light grey areas over Scandinavia and Scotland represent Early Holocene ice cover. Dark grey areas are excluded from the analysis due to low site density. Grey crosses represent the location of pollen sites used for the interpolation.

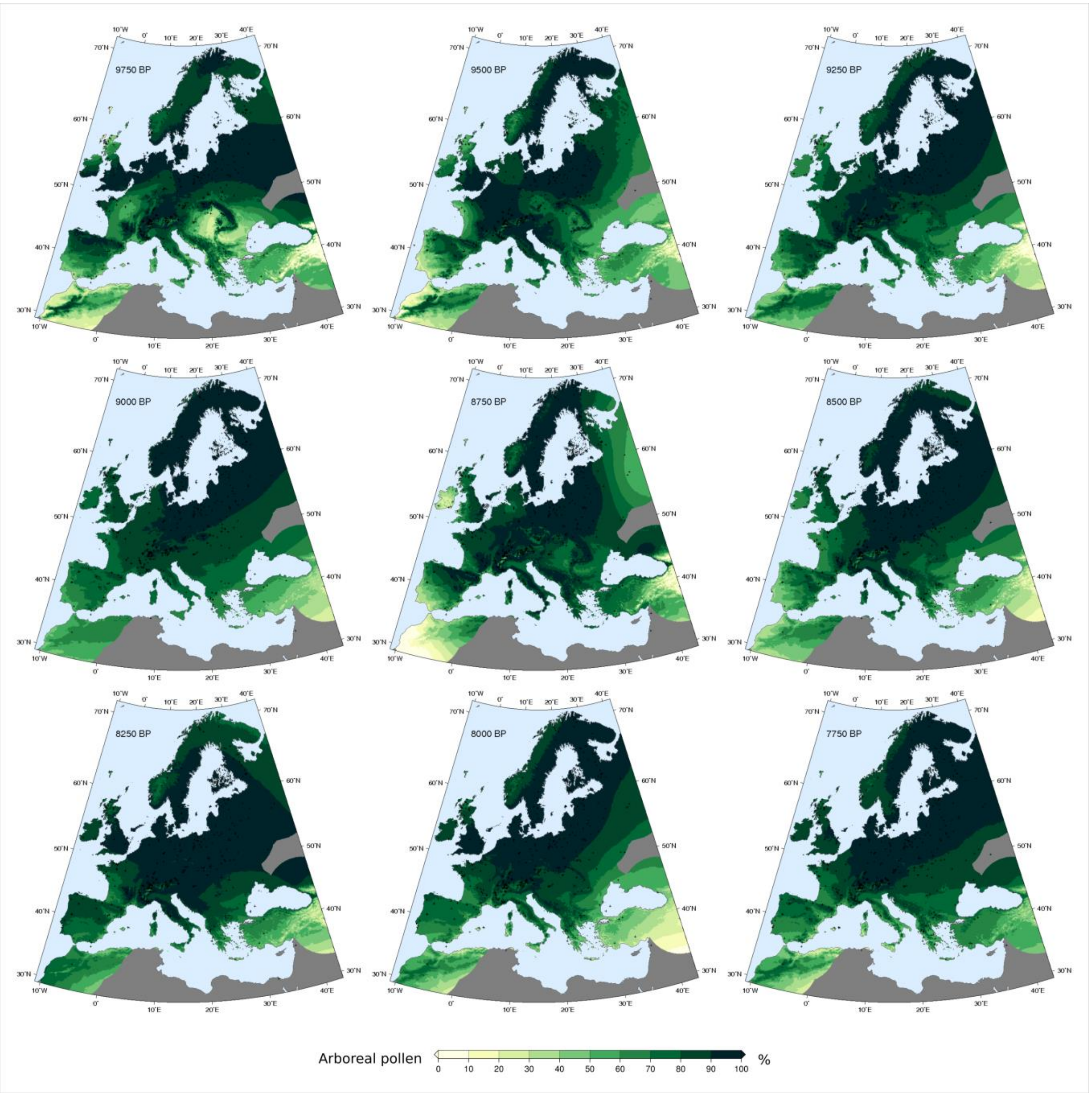

(continues from fig. S5)

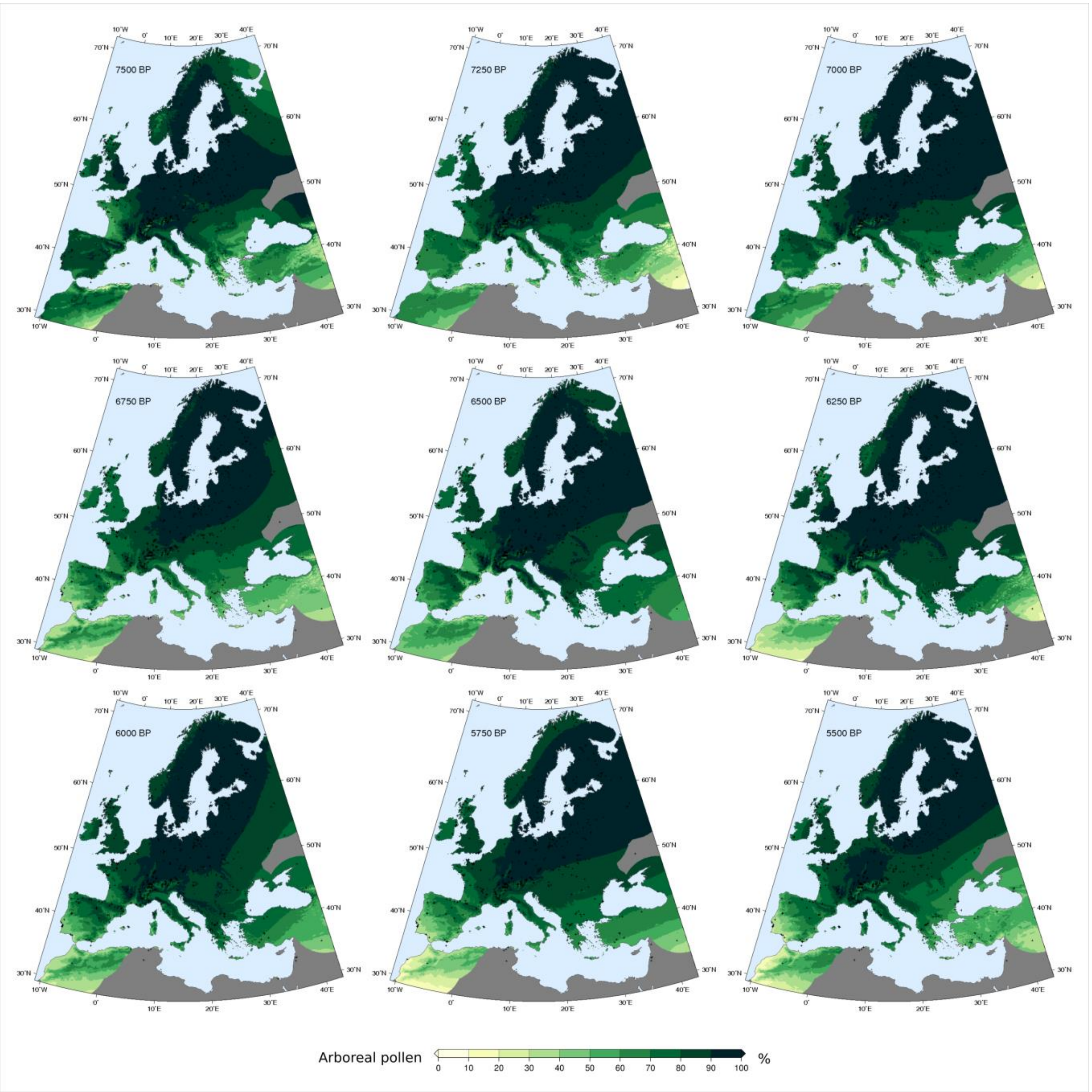

(continues from fig. S5)

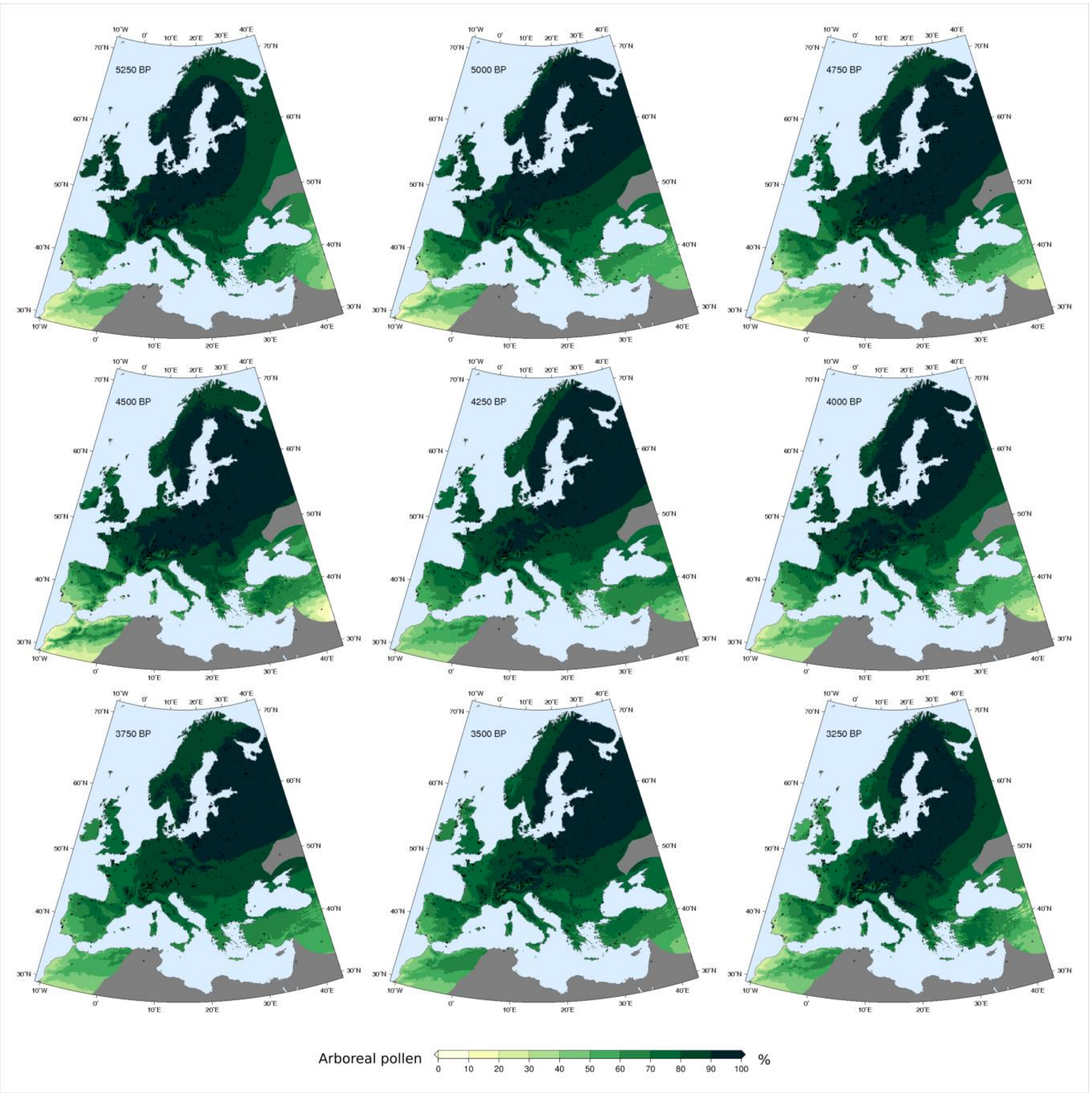

(continues from fig. S5)

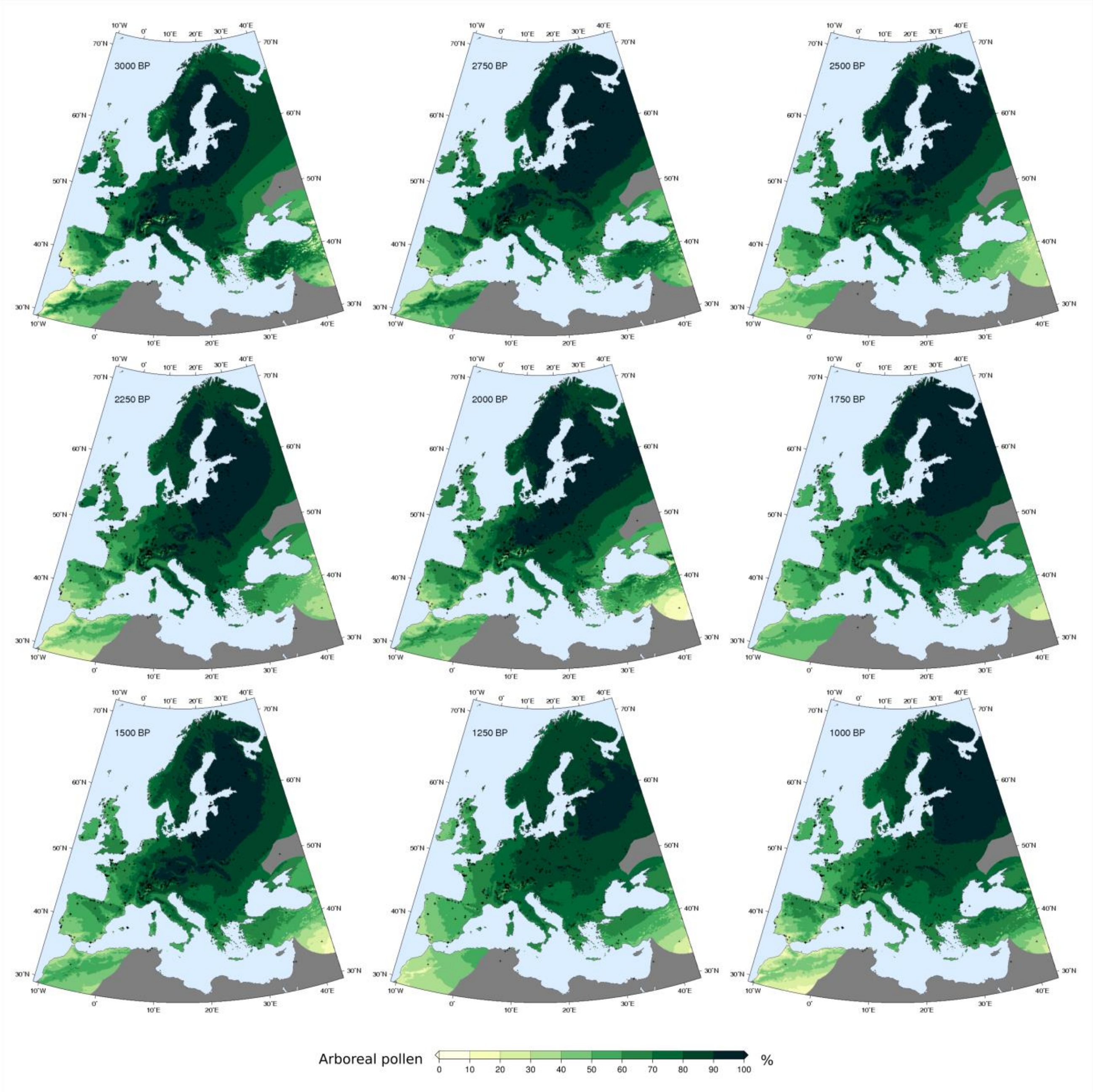

(continues from fig. S5)

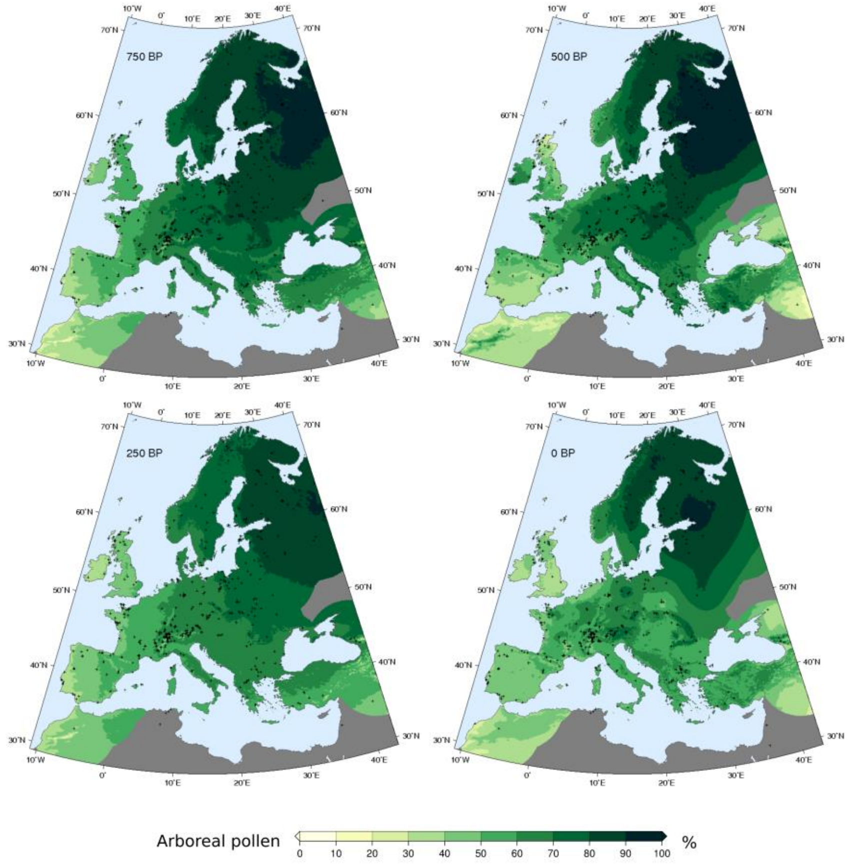

(continues from fig. S5)

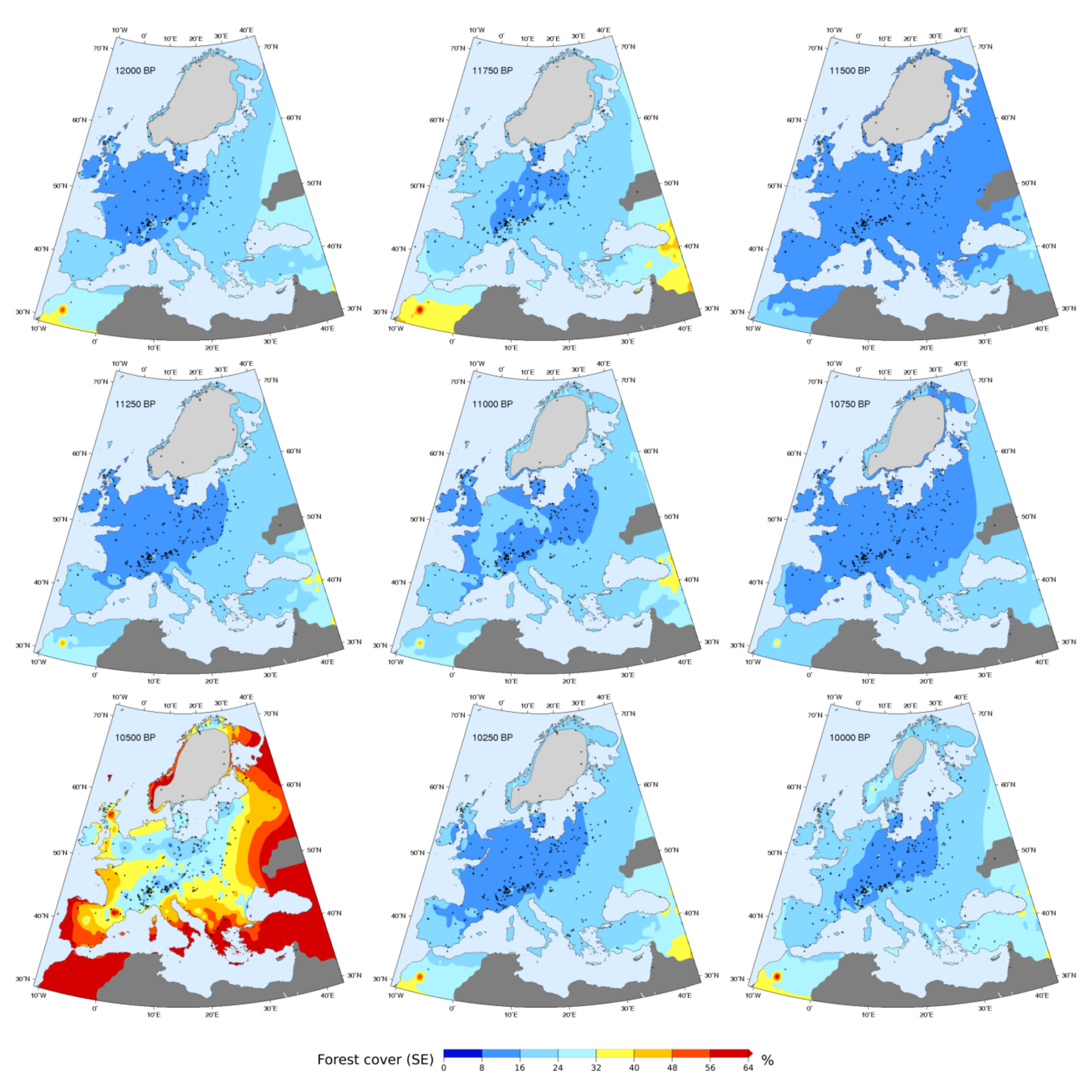

Figure S6. Standard Error estimates for the forest cover reconstruction. Light grey areas over Scandinavia and Scotland represent Early Holocene ice cover. Dark grey areas are excluded from the analysis due to low site density. Grey crosses represent the location of pollen sites used for the interpolation.

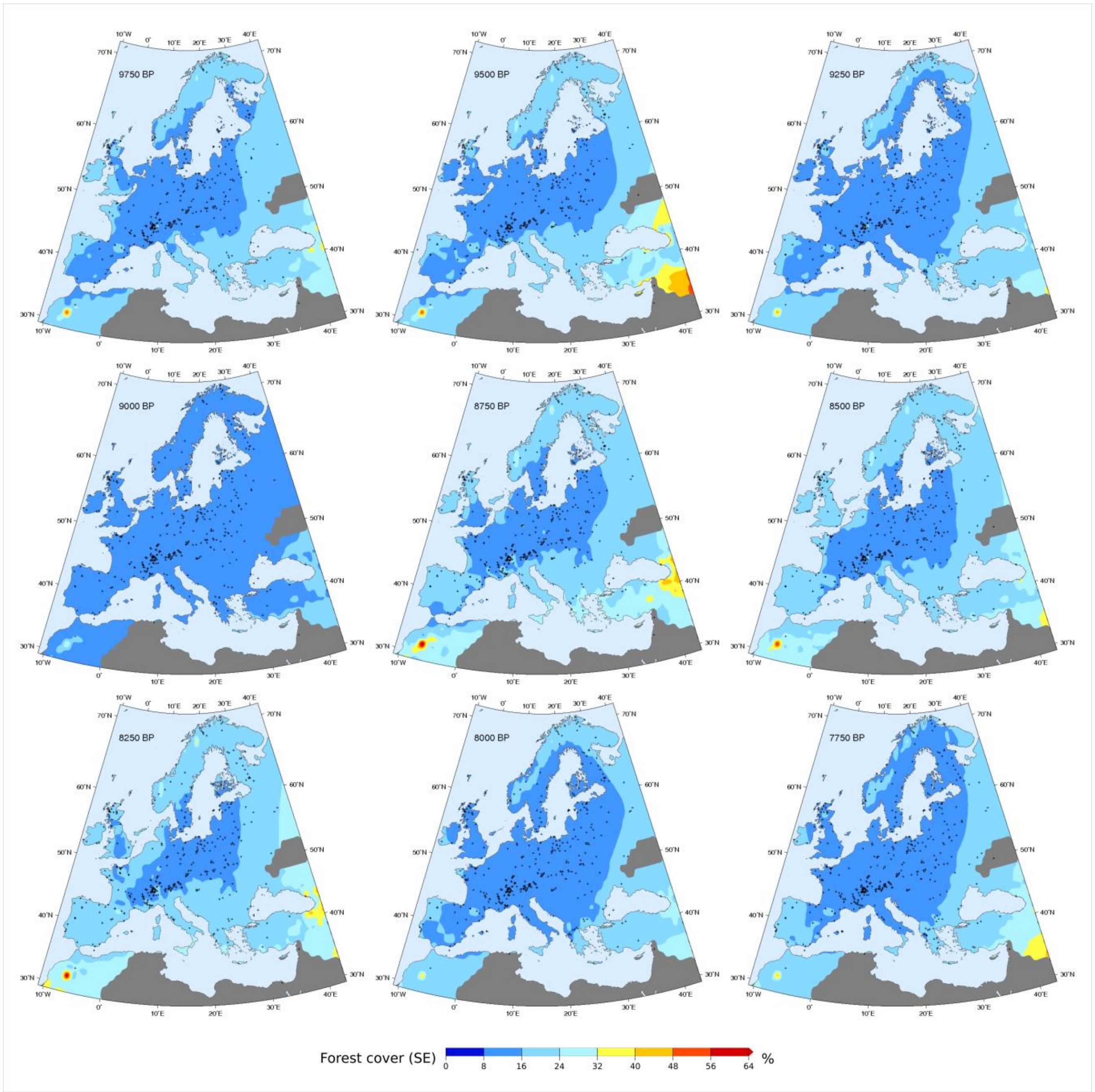

(continues from fig. S6)

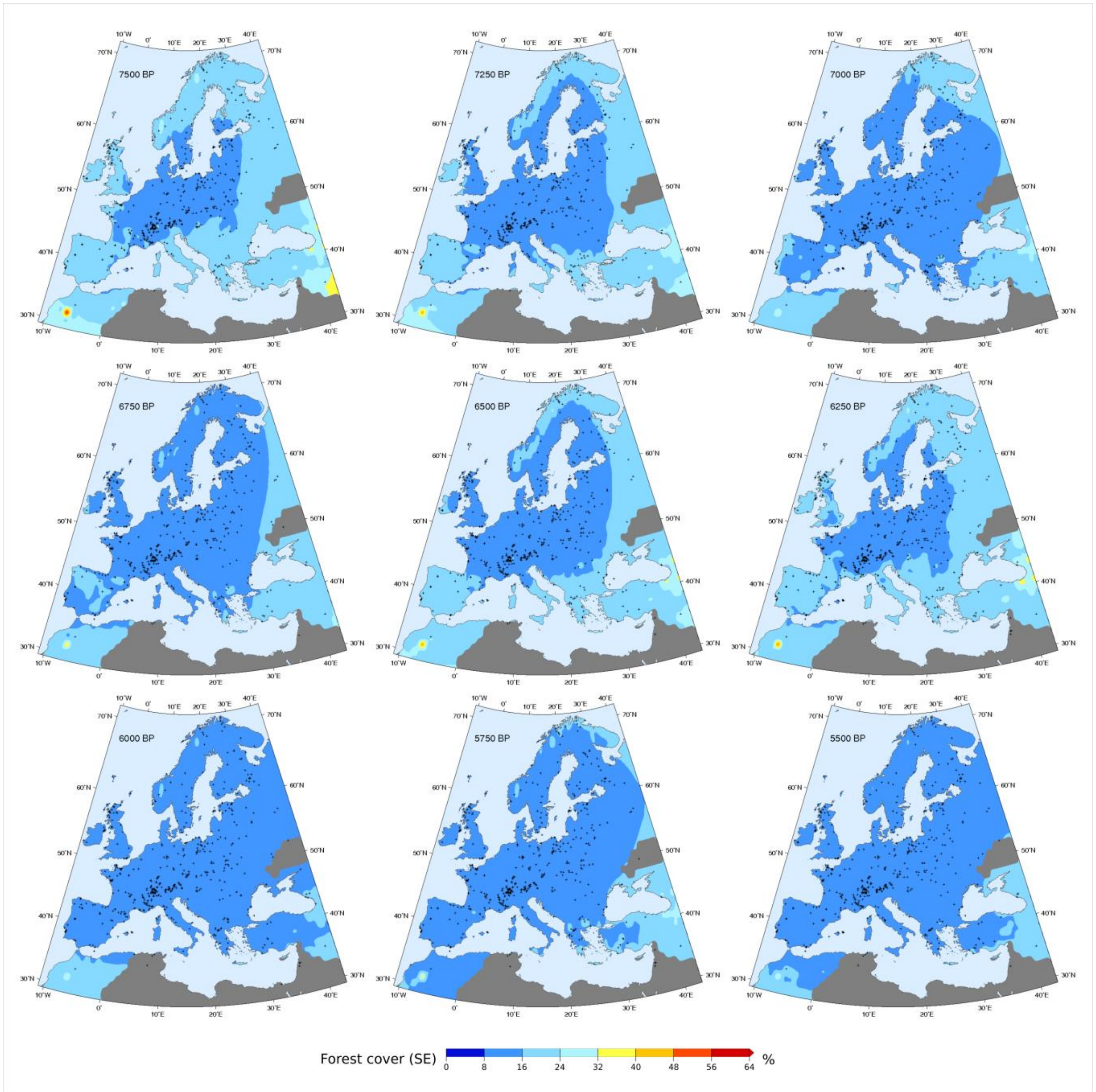

(continues from fig. S6)

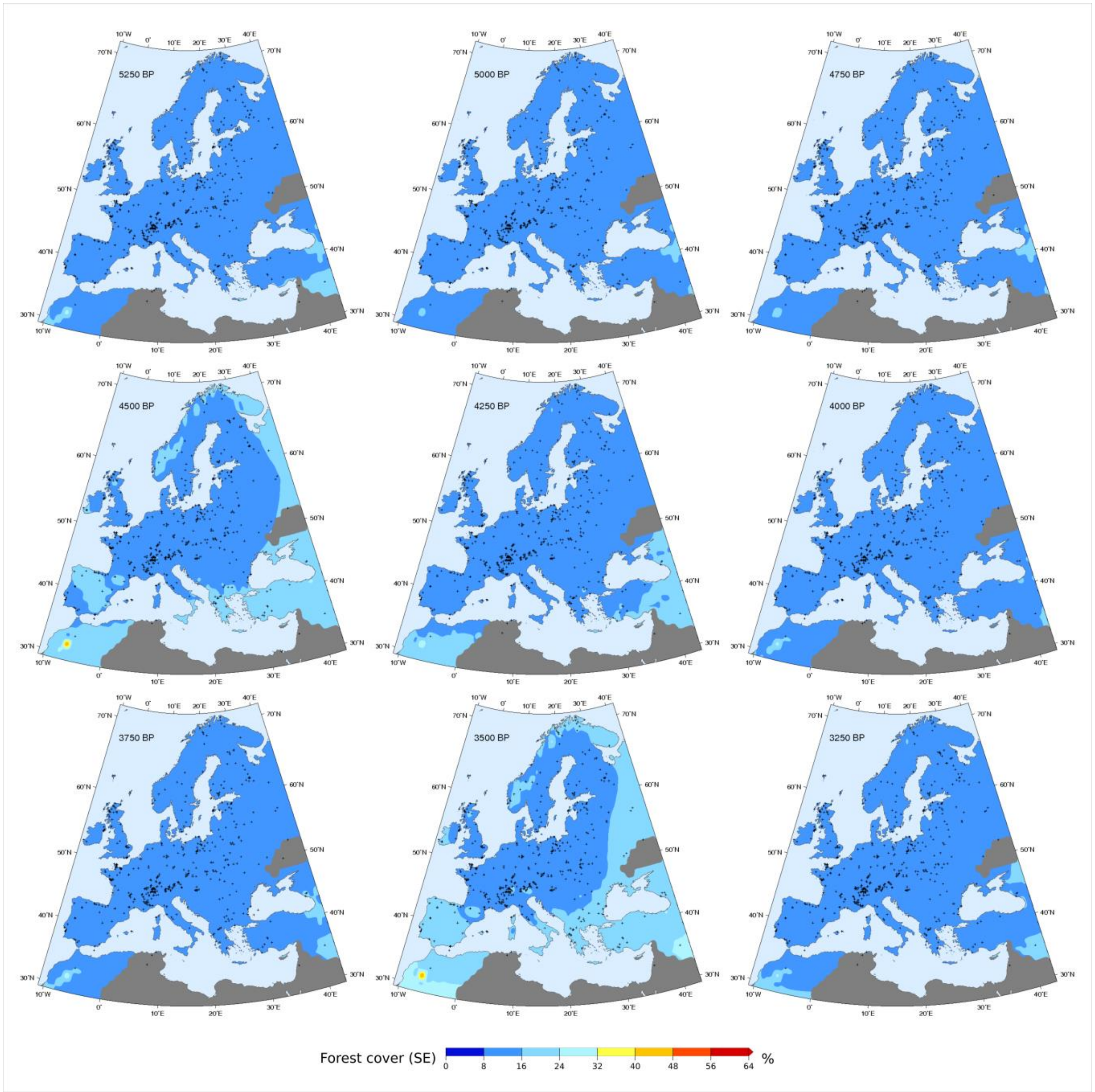

(continues from fig. S6)

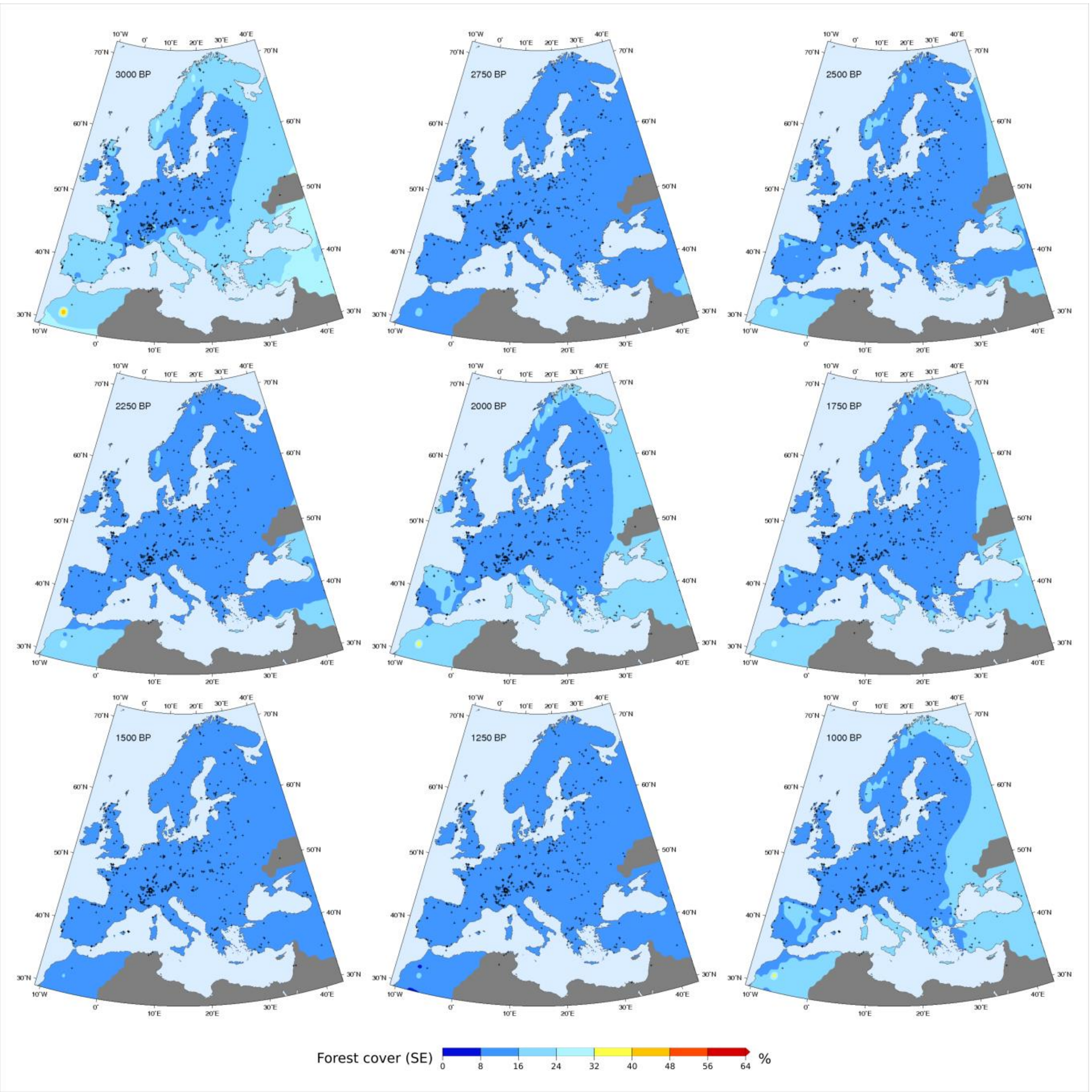

(continues from fig. S6)

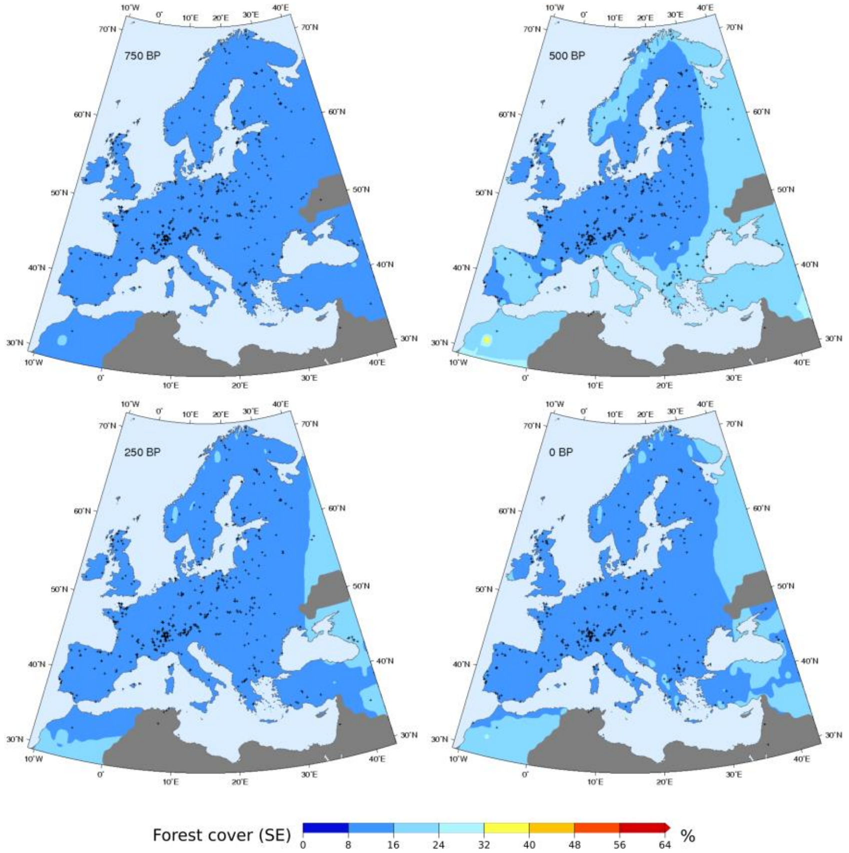

(continues from fig. S6)

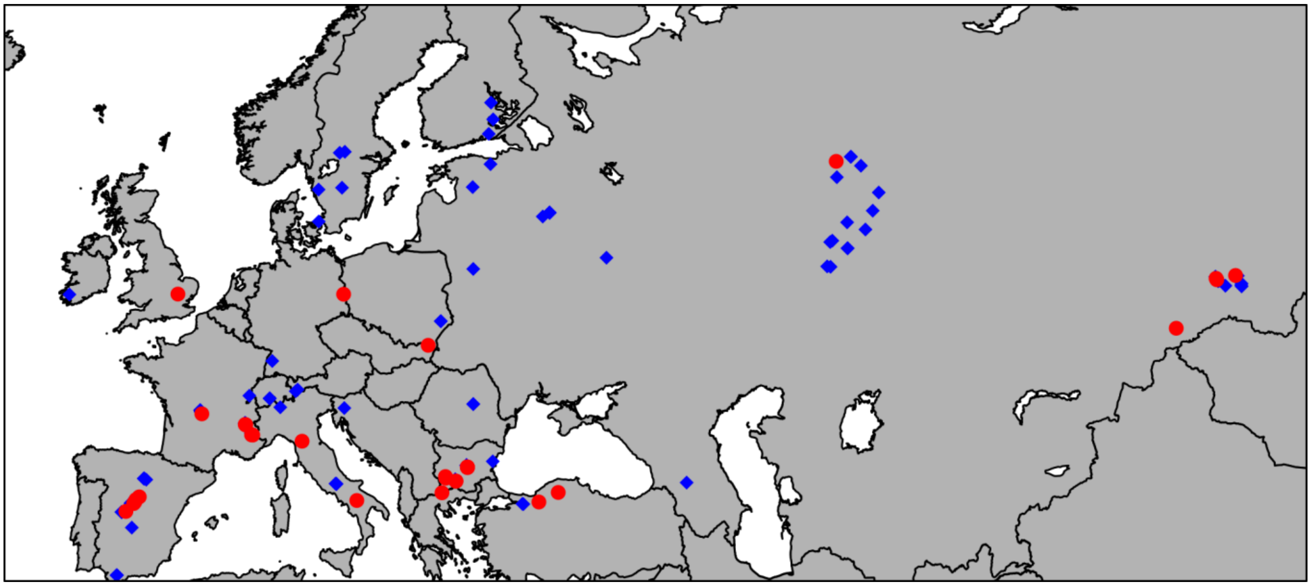

Figure S7. Map displaying sites with modern tree cover higher than 80%. Blue diamonds: sites with residual values higher than -40. Red circles: sites with residual values lower than -40.

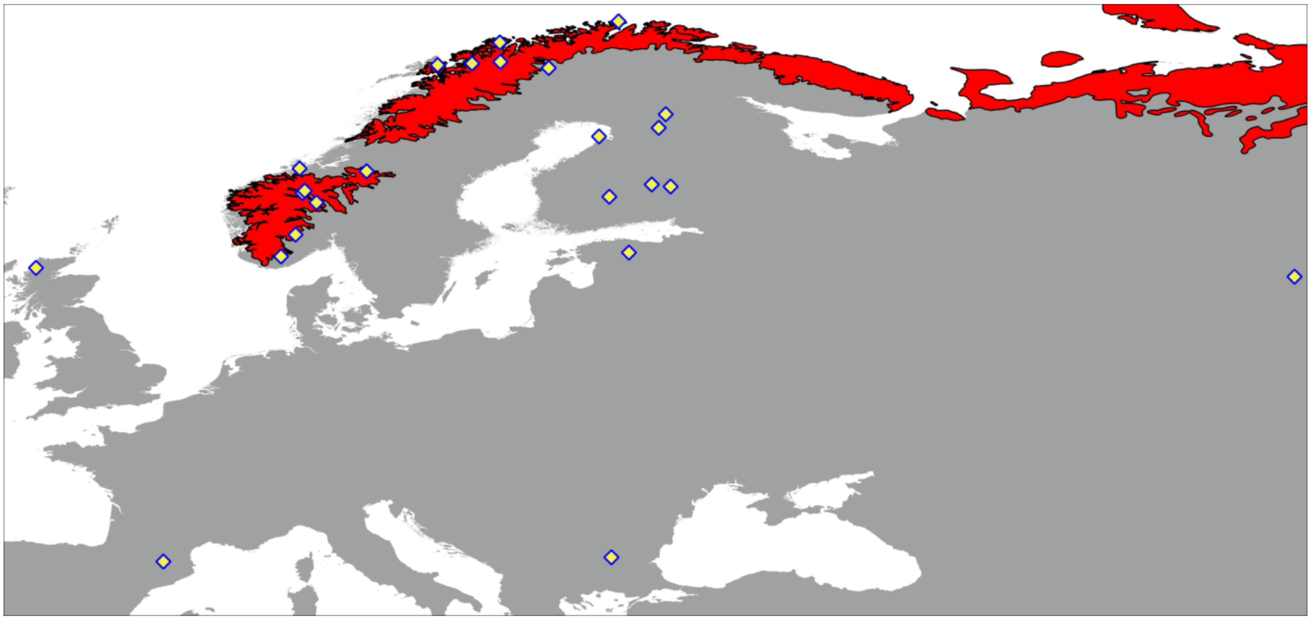

Figure S8. Location of the closest modern analogues (yellow diamonds, single closest analogue for each fossil sample) for the five sites involved in the MAT-REVEALS comparison (fig. 4) within the 12,000 – 10,000 BP period. The extent of the tundra biome (Olson et al., 2001) is highlighted in red.

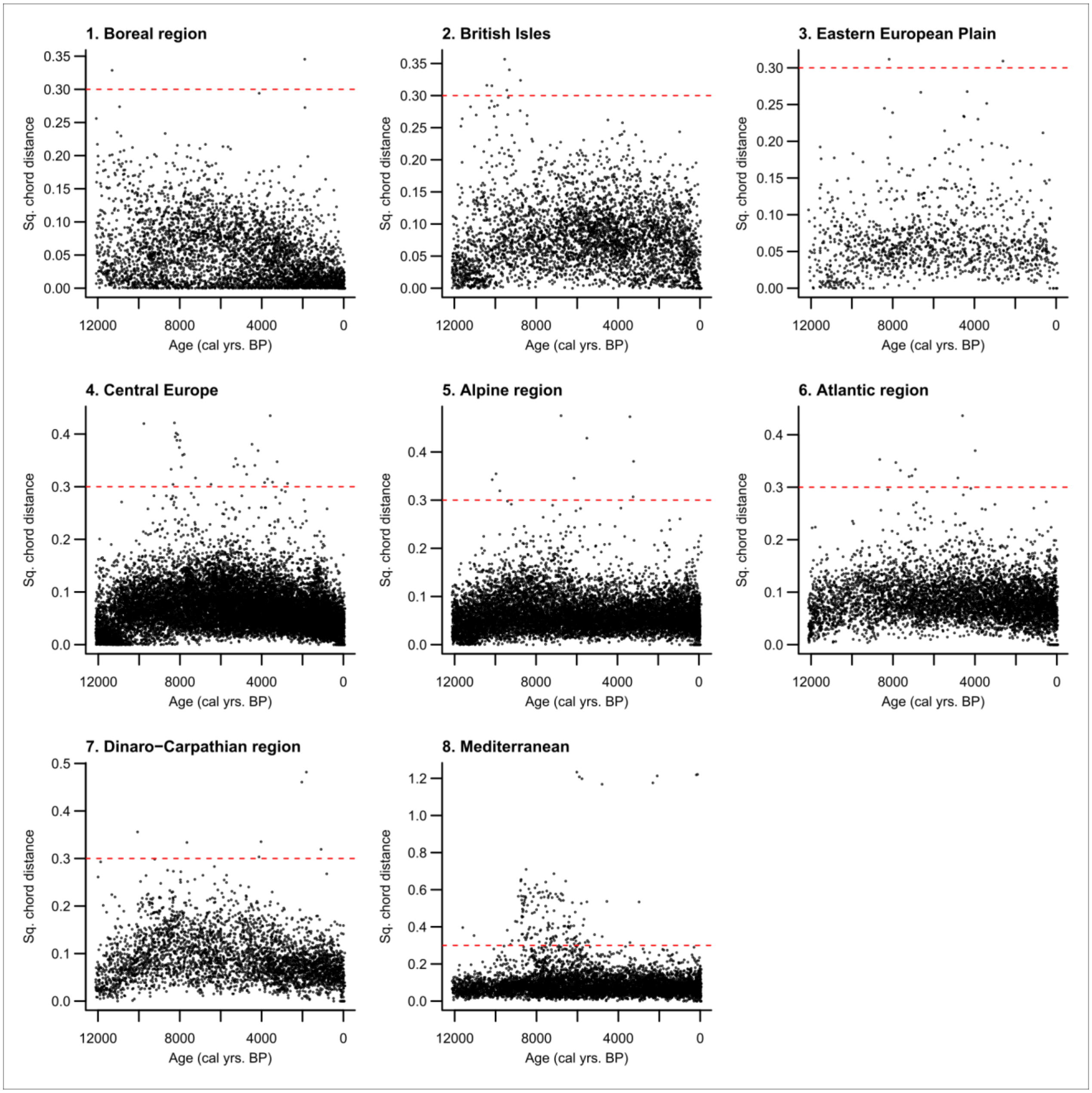

Figure S9. Minimum squared chord distances for each region across the whole fossil data set using the PFT approach. The horizontal dashed red line marks the 0.3 value used as a threshold in the present study following Huntley (1990). The Mediterranean region emerges as having the highest occurrence of no-analogue samples, likely connected to the low representation of extensive Mediterranean forests in the training data set.

1.2 Supplementary Tables

Pollen Taxa	PFT	Code
Larix	Boreal summergreen	bs
Betula	Boreal summergreen arctic-alpine	bs/aa
Picea, *Pinus* subgen. *Haploxylon*	Boreal evergreen conifer	bec
Abies	Boreal evergreen/cool-temperate conifer	bec/ctc
Cedrus, *Taxus*	Intermediate temperate conifer	ctc1
Juniperus, *Pinus* subgen. *Diploxylon*	Eurythermic conifer	ec
Alnus, *Salix*	Temperate/boreal summergreen/arctic-alpine	ts/bs/aa
Populus	Temperate/boreal summergreen	ts/bs
Acer, *Fraxinus excelsior*, *Quercus* (deciduous)	Temperate summergreen	ts
Carpinus, *Ulmus*, *Corylus*, *Fagus*, *Frangula*, *Tilia*,	Cool-temperate summergreen	ts1
Castanea, *Platanus*, *Ostrya*, *Fraxinus ornus*, *Vitis*, *Juglans*	Warm-temperate summergreen	ts2
Quercus (evergreen)	Warm-temperate broad-leaved evergreen	wte
Buxus, *Hedera*, *Ilex*	Cool-temperate broad-leaved evergreen	wte1
Acacia, *Cistus*, *Rhus*, *Myrtus*, *Olea*, *Phillyrea*, *Pistacia*, *Ceratonia*	Warm-temperate sclerophyll trees/shrub	wte2
Hippophae, *Polygonum*	Cold grass shrub	cgs
Fabaceae, *Zizyphus,* Scrophulariaceae, *Ephedra fragilis,* Brassicaceae, Crassulaceae	Warm grass shrub	wgs
Apiaceae, Asteraceae, *Armeria,* Boraginaceae, Campanulaceae, Caryophyllaceae, *Centaurea,* Dipsacaceae, *Helianthemum, Plantago,* Plumbaginaceae, *Ranunculus,* Rosaceae, Rubiaceae, *Rumex, Sanguisorba, Thalictrum*	Steppe forb/shrub	sf
Artemisia, Chenopodiaceae	Steppe/desert forb/shrub	sf/df
Ephedra, Zygophyllaceae	Desert forb/shrub	df
Alnus fruticosa, Betula nana, Saxifraga, Empetrum, Dryas, Rhododendron, Vaccinium	Arctic-alpine dwarf shrub	aa
Poaceae	Grass	g
Ericaceae, *Calluna*	Heath	h

Table S1. Conversion of pollen counts into Plant Functional Types (PFTs) taken from Peyron et al. (1998). Pollen taxa within each pollen sample are grouped in PFTs according to shared climatic ranges and biological traits. Some taxa can belong to multiple PFTs. The complete taxa-to-PFTs procedure is described in Peyron et al. (1998).

Biome	PFT combination
Cold deciduous forest	bs+bs/aa+ts/bs/aa+ts/bs+h
Taiga	bs+bs/aa+bec+bec/ctc+ts/bs/aa+ts/bs+ec+h
Cold mixed forest	bs+bs/aa+bec/ctc+ctc1+ts/bs/aa+ts/bs+ts1+ec+h
Cool conifer forest	bs+bs/aa+bec+bec/ctc+ts/bs/aa+ts/bs+ts1+ec+h
Temperate deciduous	bs+bs/aa+bctc*+ctc1+ts/bs/aa+ts/bs+ts**+ts1+ts2+wte1+ec+h
Cool mixed forest	bs+bs/aa+bec+bec/ctc+ts/bs/aa+ts/bs+ts+ts1+ts2+ec+h
Warm mixed forest	ts/bs/aa+ts/bs+ts+ts1+ts2+wte+wte1+ec+h
Xerophytic wood/scrub	wte+wte2+ec+g
Desert (hot or cold)	df+sf/df
Cool steppe	cgs+sf+sf/df+g
Warm steppe	wgs+sf+sf/df+g
Tundra	bs/aa+ts/bs/aa+aa+h+g

Table S2. Biome assignment based on PFT scores as reported by Peyron et al. (1998). Biome scores for each sample are calculated by summing all the PFTs belonging to each biome. PFT assignment follows tab. S1. The biome having the highest score is then assigned to the corresponding sample. The complete taxa-to-PFTs procedure is described in Peyron et al. (1998). *=the presence of code *bctc* in Peyron et al (1998) is likely a typo, as it does not correspond to any PFT described in the paper. In the present paper, it was replaced with *bec/ctc* (i.e. *Abies*, tab. S1) following Prentice et al. (1996), which include *Abies* in the Temperate Deciduous biome. **= Peyron et al. (1998) do not include the *ts* PFT in the Temperate Deciduous biome. This missing PFT is likely a typo, as *ts* includes the taxon *Quercus* (deciduous), which is an important component of the Temperate Deciduous biome. In the present paper, we included the *ts* PFT in the composition of the Temperate Deciduous biome following Prentice et al. (1996).

Biomes	Initial PFT	Final PFT
Cold deciduous forest and Taiga	bs/aa, ts/bs/aa, ts/bs	bs
	ec	bec
Cold mixed forests and Cool conifer forests	bs/aa	bs
	ts/bs/aa, ts/bs	ts/bs
	ec	bec/ctc
Temperate deciduous and Cool mixed forest	bs/aa	bs
	ts/bs/aa, ts/bs	ts
	ec	bec/ctc
Warm mixed forest	ts/bs/aa, ts/bs	ts
	ec, h	wte
Xerophytic woods/scrubs	ec	wte
Tundra	bs/aa, ts/bs/aa, h, g	aa
Cool steppe	sf, sf/df	cgs
	g	aa
Warm steppe	sf, sf/df	wgs
	g	cgs
Cold desert and hot desert	sf/df	df

Table S3. PFT refinement based on biome scores as presented by Peyron et al. (1998). Some of the initial PFT scores defined in tab. S1 are reassigned according to the biome selected in tab. S2 (initial PFTs changed into final PFTs). This step refines the attribution of certain taxa to more specific PFTs. As an example, in tab. S1 *Betula* is assigned to PFT *bs/aa*. If the overall pollen content places a sample in the tundra biome, then any *Betula* pollen in that specific sample is reassigned to PFT *aa*. The complete taxa-to-PFTs procedure is described in Peyron et al. (1998).

Site	Period	Difference MAT-REVEALS (proportions of forest cover*100)	
		Uncalibrated	Calibrated
Gosciaz	Early Holocene	-16.5±11.5	2.2±16.6
	Mid-Holocene	-21.3±6.2	1.3±9.8
	Late Holocene	-12.1±11.8	8.8±11.7
Kansjon	Early Holocene	-18.7±6.3	-2.8±13.5
	Mid-Holocene	-24.3±4.6	-0.1±7.2
	Late Holocene	-16.2±11.5	5±15
Krageholmssjön	Early Holocene	-13.4±9.8	-3.2±20.8
	Mid-Holocene	-18.2±5.5	5.3±6.9
	Late Holocene	-12.8±6.8	-14.7±15.7
Raigstavere	Early Holocene	-27.4±6.8	-14.2±15.3
	Mid-Holocene	-25.4±4.7	-1.7±6.4
	Late Holocene	-17.3±11.9	4.6±10.4
Trummen	Early Holocene	-31±12.6	-15.2±22.3
	Mid-Holocene	-24.1±4.6	0.8±4.6
	Late Holocene	-10±4.9	15.3±5.3

Table S4. MAT-REVEALS comparison. Site-by-site statistical correlation values referred to fig. 4. Time-window subdivision: Late Pleistocene-Early Holocene: 11,700 – 8,100 BP. Mid- Holocene: 8,100 – 4,100 BP. Late Holocene: 4,100 - 0 BP.

1. *Abies*	24. *Fraxinus*
2. *Acer*	25. *Hedera*
3. *Alnus*	26. *Hippophae*
4. Apiaceae	27. *Ilex*
5. *Artemisia*	28. *Juglans*
6. Asteraceae	29. Lamiaceae
7. *Betula*	30. *Larix/Pseudotsuga*
8. *Carpinus*	31. *Olea/Phillyrea*
9. Caryophyllaceae	32. *Ostrya* type
10. *Castanea*	33. *Picea*
11. *Cedrus*	34. *Pinus*
12. Cerealia	35. *Pistacia*
13. Chenopodiaceae	36. Plantaginaceae
14. Cichorioideae	37. Poaceae
15. Cistaceae	38. *Quercus*
16. *Corylus*	39. Ranunculaceae
17. Cruciferae	40. Rosaceae
18. Cupressaceae	41. *Rumex*
19. Dipsacaceae	42. *Salix*
20. *Ephedra*	43. *Tilia*
21. Ericaceae	44. *Ulmus/Zelkova*
22. Fabaceae	45. Urticaceae
23. *Fagus*	46. *Vitis*

Table S5. Selection of taxa used in the taxa-based MAT test for no-analogue situations (fig.6). The wide taxonomical variability of the modern and fossil databases was simplified by grouping taxa into distinctive genera, generally following the guidelines of Beug (2004) and Moore et al. (1991). The selected pollen taxa include the most frequently occurring taxa recorded in both the modern and fossil databases. Additional, less frequent pollen taxa were also taken into account due to their potential help in discriminating between vegetation assemblages (e.g. Cedrus, Olea/Phillyrea for distinctively circum-Mediterranean communities).

Bibliography

Beug, H.-J. (2004). *Leitfaden der Pollenbestimmung für Mitteleuropa und angrenzende Gebiete*. Verlag Dr. Friedrich Pfeil.

Huntley, B. (1990). Dissimilarity mapping between fossil and contemporary pollen spectra in Europe for the past 13,000 years. *Quat. Res.* 33, 360–376.

Moore, P. D., Webb, J. A., and Collison, M. E. (1991). *Pollen analysis.* Blackwell scientific publications.

Olson, D. M., Dinerstein, E., Wikramanayake, E. D., Burgess, N. D., Powell, G. V. N., Underwood, E. C., et al. (2001). Terrestrial Ecoregions of the World: A New Map of Life on Earth. *BioScience* 51, 933–938.

Peyron, O., Guiot, J., Cheddadi, R., Tarasov, P., Reille, M., de Beaulieu, J.-L., et al. (1998). Climatic Reconstruction in Europe for 18,000 YR B.P. from Pollen Data. *Quat. Res.* 49, 183–196. doi:10.1006/qres.1997.1961.

Prentice, C., Guiot, J., Huntley, B., Jolly, D., and Cheddadi, R. (1996). Reconstructing biomes from palaeoecological data: a general method and its application to European pollen data at 0 and 6 ka. *Clim. Dyn.* 12, 185–194.
